# Cdk1 inactivation induces post-anaphase-onset spindle migration and membrane protrusion required for extreme asymmetry in mouse oocytes

**DOI:** 10.1038/s41467-018-06510-9

**Published:** 2018-10-02

**Authors:** Zhe Wei, Jessica Greaney, Chenxi Zhou, Hayden A. Homer

**Affiliations:** The Christopher Chen Oocyte Biology Research Laboratory, Centre for Clinical Research, Faculty of Medicine, The University of Queensland, Herston, QLD 4029 Australia

## Abstract

Female meiotic divisions are extremely asymmetric, producing large oocytes and small polar bodies (PBs). In mouse oocytes, the spindle relocates to the cortex before anaphase of meiosis I (MI). It is presumed that by displacing the future midzone, pre-anaphase spindle repositioning alone ensures asymmetry. But how subsequent anaphase events might contribute to asymmetric PB extrusion (PBE) is unknown. Here, we find that inactivation of cyclin-dependent kinase 1 (Cdk1) induces anaphase and simultaneously triggers cytoplasmic formin-mediated F-actin polymerisation that propels the spindle into the cortex causing it to protrude while anaphase progresses. Significantly, if post-anaphase-onset spindle migration fails, protrusion and asymmetry are severely threatened even with intact pre-anaphase migration. Conversely, post-anaphase migration can completely compensate for failed pre-anaphase migration. These data identify a cell-cycle-triggered phase of spindle displacement occurring after anaphase-onset, which, by inducing protrusion, is necessary for extreme asymmetry in mouse oocytes and uncover a pathway for maximising unequal division.

## Abstract (web summary)

Female meiotic divisions are asymmetric with the formation of large oocytes and small polar bodies, thought to result from cortical spindle placement before anaphase. The authors find that Cdk1 inactivation triggers F-actin dependent post-anaphase spindle migration, resulting in cortical protrusion.

## Introduction

Highly asymmetric divisions in mouse oocytes represent the culmination of a number of coordinated steps. First, bipolar spindles assemble in the absence of canonical centrosomes close to the oocyte centre^1^ and migrate to the cortex before anaphase-onset^2–6^. Anaphase then occurs when separase is activated to cleave the molecular glue, cohesin, holding chromosomes together^7^. Separase activation requires anaphase-promoting complex (APC)-mediated destruction of the separase inhibitor, securin, and inactivation of the master cell-cycle kinase, cyclin-dependent kinase 1 (Cdk1), brought about through APC-mediated proteolysis of the Cdk1 co-activator, cyclin B1^8,9^. Finally, formation of the polar body (PB) incorporates membrane outpocketing (or protrusion), membrane furrowing and cytokinesis^10,11^. The actomyosin cytoskeleton is known to be important for events on either side of protrusion, that is, pre-anaphase spindle migration and contractile ring formation that directs furrowing^2–6,10–13^. Although many such details occurring around the time of PB extrusion (PBE) in mouse oocytes have been worked out, surprisingly little is known about how protrusion comes about and how various components are integrated in time and space and with key cell-cycle regulators to efficiently achieve division asymmetry.

In *Xenopus* and *Caenorhabditis elegans* oocytes, protrusion involves relaxation of the cortex overlying the spindle (polar relaxation) either due to increased dynamic actin behaviour (*Xenopus*)^14^ or to localised depletion of F-actin (*C. elegans*)^15^. In contrast, around the time of anaphase in meiosis I (MI) mouse oocytes, actin becomes more stable and less dynamic in the polar region^3^, raising the question of how a localised cortical region that does not favour relaxation is induced to protrude^11^. Moreover, contrasting opinions exist regarding how protrusion occurs in mouse oocytes and its relationship to the cell cycle. Some findings suggest that protrusion arises during metaphase I with a spindle oriented parallel to the cortex that then rotates through 90° following anaphase^16^ akin to what has been described for calcium-triggered second PB formation during MII^17^. Parallel orientation of the spindle prior to anaphase seems at odds, however, with the pole-first migration that occurs prior to this and other data indicating that protrusion in MI is associated with anaphase and a spindle oriented perpendicular to the cortex^18^.

Here, by studying peri-anaphase events in MI mouse oocytes at high temporal resolution, we uncover how a key element in PB formation, protrusion, comes about and how it is coordinated with cell-cycle regulators.

## Results

### Details of anaphase during MI in mouse oocytes

During MI, the spindle migrates towards the cortex in the lead up to anaphase and first PBE. To determine the timing of these events in our system, we undertook timelapse fluorescence imaging of spindles and chromosomes labelled using silicon rhodamine (SiR)-Tubulin (used previously in mouse oocytes^19,20^) and histone H2B-mCherry, respectively, from entry into MI, marked by germinal vesicle breakdown (GVBD). We captured images at 30-min intervals and found that a bipolar spindle assembled over 3–4 h, migrated to the cortex between 4–7 h post-GVBD after which anaphase and PBE ensued between 7–9 h post-GVBD (Supplementary Fig. 1 and Supplementary Movie 1).

**Fig. 1:**
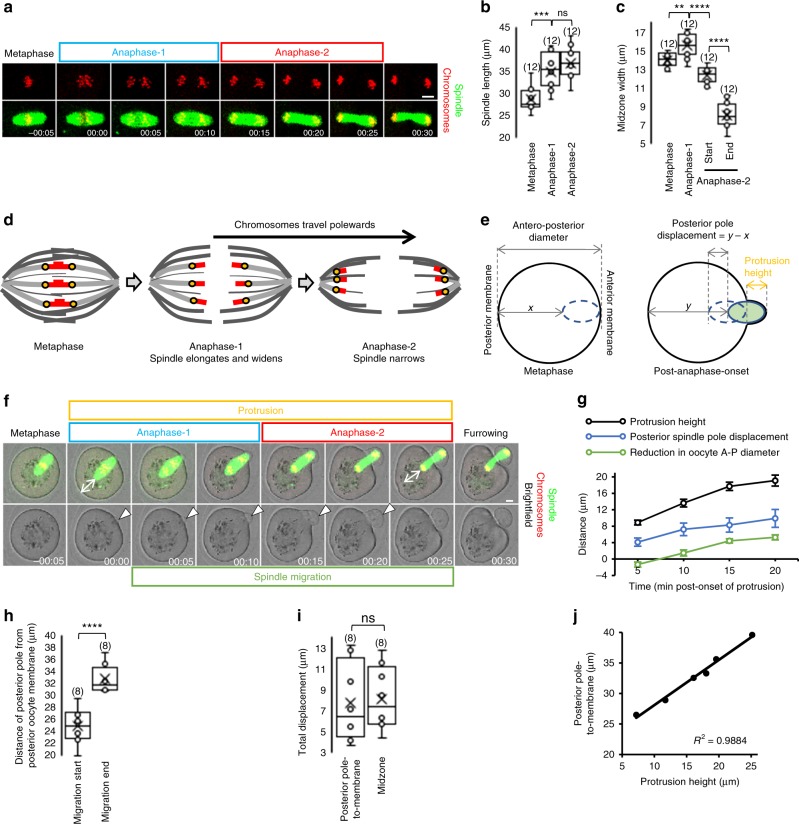
Spindle migration following anaphase-onset induces protrusion. **a**–**c** Stages of anaphase (**a**) and quantification of stage-specific spindle dimensions (**b**, **c**) derived from live oocytes with fluorescently labelled spindles and chromosomes. One-way ANOVA used for statistical analysis. **d**, **e** Schematics depicting stages of anaphase (**d**) and measurements (**e**). **f** Example of protrusion (white arrowheads) and spindle migration into the membrane occurring after anaphase-onset in a live oocyte (see Supplementary Movie 3). **g**–**i** Quantification of protrusion height, spindle displacement and oocyte dimensions (**g**, *n* = 12, error bars are mean ± SEM) (see **e**); total posterior pole displacement (**h**) and comparison with total midzone displacement (**i**) between anaphase-onset and anaphase-completion. Two-tailed Student’s *t* test used for statistical analysis. **j** Correlation between spindle migration and protrusion height (*n* = 12). Times in panels are hours:minutes relative to anaphase-onset. Scale bars, 10 µm. Box plots depict median (horizontal line), mean (crosses), 25th and 75th percentiles (boxes) and 5th and 95th percentiles (whiskers). Oocyte numbers are shown in parenthesis. *P* values represented as **P < 0.01, ***P < 0.001 and ****P < 0.0001, ns denoted *P* > 0.05

Image capture at 15–30-min intervals, as is often used for studying mouse oocytes, did not allow sufficiently detailed analysis of anaphase and associated membrane changes. We therefore acquired images at 5-min intervals starting from 6 h post-GVBD. Anaphase began with the earliest sign that chromosome pairs were beginning to separate (evident as the first poleward dispersal of chromosomes after metaphase; see Fig. 1a, frame 00:00) and concluded when chromosomes aggregated into dense clusters at poles (see Fig. 1a, frame 00:25) but before the midzone exhibited marked constriction (see Fig. 1f, frame 00:30), representative of acute furrowing. Using these criteria, we found that anaphase lasted 25.4 ± 1.1 min (mean ± SEM) and could be divided into two components, which we term anaphase-1 and -2 (Fig. 1a). Anaphase-1 lasted 12.1 ± 0.7 min and was associated with increased spindle length and movement of chromosomes towards the poles (Fig. 1a, b, d). Chromosomes reached the poles and aggregated into condensed clusters by the end of anaphase-2, which was of similar duration to anaphase-1 (13.3 ± 0.7 min; *P* = 0.24; Student’s *t* test) and associated with narrowing of the spindle midzone (Fig. 1a, c, d). We repeated analyses using green fluorescent protein (GFP)-tagged MAP7, a microtubule marker used previously to study spindle dynamics in mouse oocytes^3,21^. We found that, during anaphase, the changes in spindle length and midzone width for MAP7-GFP-labelled spindles were indistinguishable from those of SiR-Tubulin-labelled spindles (Supplementary Fig. 2a–c and Supplementary Movie 2).

Thus, in MI mouse oocytes, chromosomes travel polewards throughout anaphase, with spindle elongation and midzone narrowing occurring during the first and second phases, respectively (Fig. 1d). As this differs from canonical anaphase-A (chromosome separation to poles) and anaphase-B (spindle elongation), we use the terms anaphase-1 and anaphase-2 (note that this is numerical 1 and 2 versus Roman numerals, I and II, which refer to first and second meiosis, respectively).

### Post-anaphase-onset migration correlates with protrusion

During MII, two empty protrusions form in relation to anaphase chromosomes, one of which forms the second PB after becoming occupied by one of the spindle poles following spindle rotation^17,18^. During MI, it is unknown whether protrusion also happens first, before ingress of the bipolar spindle. We found that, concurrent with anaphase progression, the membrane protruded as reflected by an increase in membrane height (Fig. 1e–g and Supplementary Movie 3). Significantly, the first sign of protrusion always occurred during anaphase-1 (Fig. 1f) and never before (20 of 20 oocytes) (Supplementary Fig. 3 and Supplementary Movies 4 and 5). Using a fluorescent utrophin probe (UtrCH-mCherry), which binds to and labels F-actin^3,22,23^, we confirmed that the cortex underwent changes identical to the membrane beginning after anaphase-onset (Supplementary Fig. 4 and Supplementary Movie 6).

During anaphase, and while the outpocketing enlarged, the leading pole of the anaphase spindle became increasingly enveloped by the protrusion (Fig. 1f and Supplementary Fig. 4). Indeed, by the end of anaphase-2, the maximal protrusion height (~18 µm) was half of the total length of the spindle (~37 µm; Fig. 1b, e–g and Supplementary Fig. 4) consistent with half of the anaphase spindle becoming enclosed within the protrusion. Thus, following migration to the cortex, the spindle does not enter a pre-formed cortical protrusion. Instead, protrusion of the cortex/membrane begins after anaphase-onset and enlarges as anaphase progresses.

It is presumed that protrusion is mostly accounted for by anaphase spindle extension. However, this was clearly not the case since total anaphase spindle elongation was 8.2 ± 0.4 µm (Fig. 1b) and only roughly half of this or 4–5 µm would extend towards the protrusion. We did observe an overall retraction of the oocyte’s antero-posterior diameter that accounted for a further 5.3 ± 0.6 µm of protrusion height (Fig. 1g) altogether leaving ~8–9 µm still unaccounted for.

Unexpectedly, between anaphase-onset and completion of protrusion, the lagging pole and the midzone of the spindle travelled almost exactly this distance (~8–9 µm) away from the posterior cortex (Fig. 1e–i). This was a substantial travel distance representing 12.1 ± 1.9% of the pre-anaphase oocyte diameter, over two-fold greater than the displacement reported in *C. elegans* embryos (4.5%)^24^. We also found that spindle displacement correlated strongly with protrusion size (Fig. 1j). Notably, MAP7-GFP-labelled spindles also migrated a very similar distance post-anaphase-onset (8.9 µm) in association with membrane protrusion (Supplementary Fig. 2c, d and Supplementary Movie 2). Collectively, these data support that the spindle migrates again after anaphase-onset and could be critical for protrusion.

### Cdk1 inactivation triggers post-anaphase-onset migration

Since the second phase of spindle migration occurred strictly after anaphase-onset, we asked whether mechanisms that initiate anaphase might also induce spindle migration/protrusion. Anaphase requires APC-mediated proteolysis of securin and cyclin B^8,9,25^ and inactivation of Cdk1^26^. To test the importance of Cdk1 inactivation, we used the potent Cdk1 inhibitor, flavopiridol, at the same dose (5 µM) used previously in mammalian somatic cells^27^ and mouse oocytes^28,29^. Under our conditions, 5 µM of flavopiridol completely abolished GVBD (*n* = 30), confirming highly effective inhibition of Cdk1 (Supplementary Fig. 5).

**Fig. 2:**
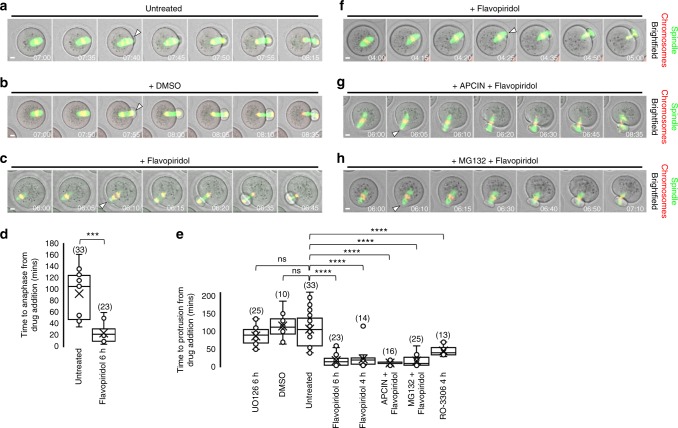
Protrusion requires Cdk1 inactivation but not proteolysis. **a**–**e** Flavopiridol-induced Cdk1 inactivation accelerates anaphase and protrusion-onset. Oocytes were untreated (**a**; see Supplementary Movie 7) or treated with DMSO (**b**; see Supplementary Movie 8) or flavopiridol (**c**; see Supplementary Movie 9) from 6 h post-GVBD. Quantification of time of anaphase-onset (**d**) and protrusion-onset (**e**). Two-tailed Student’s *t* test (**d**) or one-way ANOVA (**e**) used for statistical analysis. *P* values represented as ***P < 0.001 and ****P < 0.0001, ns denoted *P* > 0.05. Box plots depict median (horizontal line), mean (crosses), 25th and 75th percentiles (boxes) and 5th and 95th percentiles (whiskers). **f**–**h** Flavopiridol induces protrusion despite lack of proteolysis. Oocytes were treated either with flavopiridol prior to proteolysis onset (see Supplementary Figure 6) (**f**; see Supplementary Movie 10) or with the APC-inhibitor, APCIN, followed by flavopiridol (**g**; see Supplementary Movie 11) or the 26S proteasome inhibitor, MG132, followed by flavopiridol (**h**; see Supplementary Movie 12). Anaphase failure reflects lack of proteolysis resulting in chromosome entrapment within the furrow. Times in panels are hours:minutes post-GVBD. Scale bars, 10 µm. White arrowheads indicate protrusion-onset. Oocyte numbers are shown in parentheses from a minimum of three independent experiments

We first treated oocytes at 6 h post-GVBD when proteolysis was beginning (Supplementary Fig. 6) and most pre-anaphase spindles had migrated to the cortex (Supplementary Fig. 1). Flavopiridol treatment at 6 h post-GVBD induced markedly earlier anaphase-onset and protrusion than either untreated or dimethyl sulphoxide (DMSO)-treated controls (Fig. 2a–e and Supplementary Movies 7–9) consistent with accelerated PBE observed previously^28^. Flavopiridol addition at 4 h post-GVBD—when a bipolar spindle had formed and spindles were nearing the cortex (Supplementary Fig. 1) but proteolysis had not yet begun (Supplementary Fig. 6) hence leaving the majority of securin intact—also induced spindle migration to the cortex and then into the membrane, and hence protrusion, in the same accelerated manner as oocytes treated at 6 h post-GVBD (Fig. 2e, f). However, owing to lack of securin destruction, chromosomes did not segregate and became trapped within the cleavage furrow (Fig. 2f and Supplementary Movie 10) showing that spindle migration/protrusion did not require anaphase.

These data suggested that Cdk1 inactivation rather than accompanying proteolysis was key for migration and protrusion. We tested this using the APC-specific inhibitor, APCIN^30^, and the 26S proteasome inhibitor, MG132, which blocked proteolysis of securin and cyclin B1 (Supplementary Fig. 6). When Cdk1 was inactivated in APCIN- or MG132-treated oocytes, protrusion was still accelerated (Fig. 2e, g, h), but because of stable securin, chromosomes again became trapped within the cleavage furrow (Fig. 2g, h and Supplementary Movies 11 and 12). These effects were specific to Cdk1 inhibition since another Cdk1 inhibitor, RO-3306^31^, also markedly accelerated protrusion (Fig. 2e), whereas protrusion onset was unaltered by inhibiting mitogen-activated protein kinase (MAPK) late in MI (Fig. 2e).

Thus, Cdk1 inactivation, but not proteolysis, is critical for inducing post-anaphase-onset migration required for protrusion. These data also show clearly that the post-anaphase-onset phase of migration that induces protrusion is not merely a passive continuation of pre-anaphase migration because, first, it requires Cdk1 inactivation and, second, metaphase spindles do not induce protrusion even after long dwell times at the cortex (Supplementary Fig. 3 and Supplementary Movies 4 and 5).

### Sustained protrusion requires an intact spindle

Consistent with F-actin being highly stable in the polar region during late MI^3^, we found that cortical F-actin thickness was greatest within the protrusion (Supplementary Fig. 7). Hence, outpocketing is unlikely to result solely from cortical relaxation and might instead require a force from within the oocyte^11^. Since protrusion is spatially restricted to the leading spindle pole and, together with spindle migration, is preceded by Cdk1 inactivation, we hypothesised that Cdk1 inactivation triggers a force that is transmitted via the spindle.

**Fig. 3:**
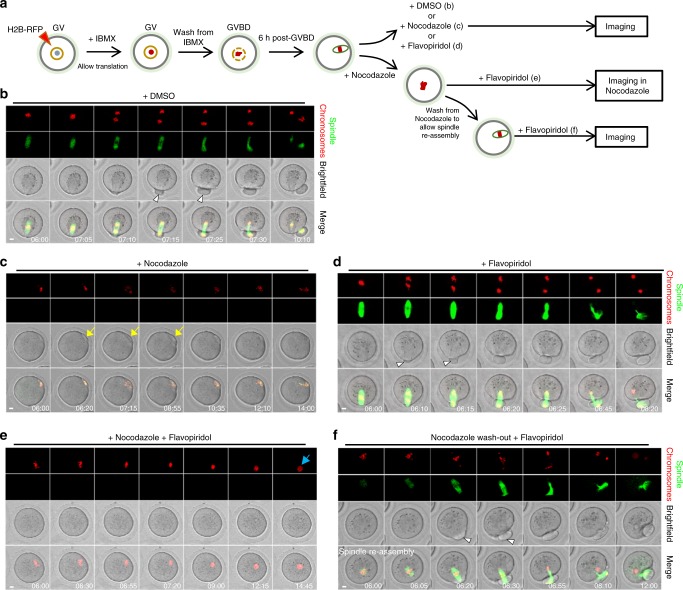
Chromosomes alone cannot induce sustained protrusions. **a** Schematic of experimental procedure. Oocytes were treated with the spindle depolymerising drug, nocodazole, DMSO or flavopiridol as indicated in the schematic at 6 h post-GVBD when most spindles were located cortically (see Supplementary Figure 1). **b**–**f** Panels of representative timelapse images corresponding to experiments shown in **a**. **b** Anaphase, protrusion and PBE in DMSO-treated oocytes (see Supplementary Movie 13). **c** Following nocodazole treatment, the spindle rapidly depolymerised leaving only chromosomes near the cortex. Peripherally located chromosomes never induced a sustained protrusion in nocodazole-treated oocytes (*n* = 16). In some oocytes (3 of the 16), small transient protrusions were observed (yellow arrow) that receded and never produced PBs (see Supplementary Movie 14). **d**–**f** Chromosomes alone could not induce protrusion if Cdk1 was inactivated with flavopiridol. As before (Fig. 2c–e), flavopiridol rapidly induced protrusion with intact spindles (white arrowheads) (**d**; see Supplementary Movie 15). In contrast, following nocodazole treatment, flavopiridol failed to induce protrusion (*n* = 21; **e**; see Supplementary Movie 16). Chromosomes eventually decondensed in the presence of flavopiridol (blue arrow). Treating oocytes with flavopiridol after washing out from nocodazole to allow a spindle to reassemble enabled sustained protrusions (white arrowhead) followed by PBE (*n* = 22; **f**; see Supplementary Movie 17). Times in panels are hours:minutes post-GVBD. Scale bars, 10 μm

If the spindle is required for force transmission, protrusion should fail without a spindle. To test this, we used nocodazole to depolymerise the spindle after it had migrated to the cortex. We found that neither a sustained protrusion nor PB formation occurred (16 of 16 oocytes), even following forced Cdk1 inactivation (21 of 21 oocytes; Fig. 3a–e and Supplementary Movies 13–16). In contrast, if oocytes were washed out of nocodazole to allow spindle re-assembly, protrusions and PBs formed in relation to spindle poles shortly after Cdk1 was inactivated by flavopiridol (22 of 22 oocytes; Fig. 3f and Supplementary Movie 17).

To investigate the requirement for spindles further, we made use of the spindle collapse that eventually occurs after flavopiridol-mediated Cdk1 inactivation induces an interphase-like state^29^. We treated oocytes with flavopiridol at ~2–3 h post-GVBD, before migration to the cortex had occurred (Supplementary Fig. 1). The spindle collapsed in most oocytes prior to reaching the cortex (29 of 37 oocytes), and in these cases, a sustained protrusion was never observed (29 of 29 oocytes; Supplementary Fig. 8a and Supplementary Movie 18). In contrast, in the minority of oocytes, in which the spindle remained intact all the way to the cortex and during advancement into the membrane, a sustained protrusion followed by furrowing occurred (4 of 4 oocytes; Supplementary Fig. 8b and Supplementary Movie 19). Strikingly, we observed that in four oocytes, the spindle collapsed after protrusion began but before PBE was complete, and in all cases, the protrusion then dramatically receded (Supplementary Fig. 8c and Supplementary Movie 20).

Thus Cdk1 inactivation-induced protrusion requires an intact spindle supporting our hypothesis that protrusion involves a force transmitted via the spindle. Conversely, since sustained protrusions do not occur with chromosomes only when Cdk1 is inactivated, polar relaxation secondary to chromosome-induced cortical modifications could not be the basis for protrusion.

### Actin polymerisation is necessary for spindle migration

Next, we investigated the nature of the propulsive force that Cdk1 inactivation might initiate. In MI mouse oocytes, F-actin levels increase by ~50% around the time of anaphase^3^. Since actin polymerisation can generate pushing forces^32^, it was therefore possible that Cdk1 inactivation might trigger actin polymerisation to displace the spindle.

**Fig. 4:**
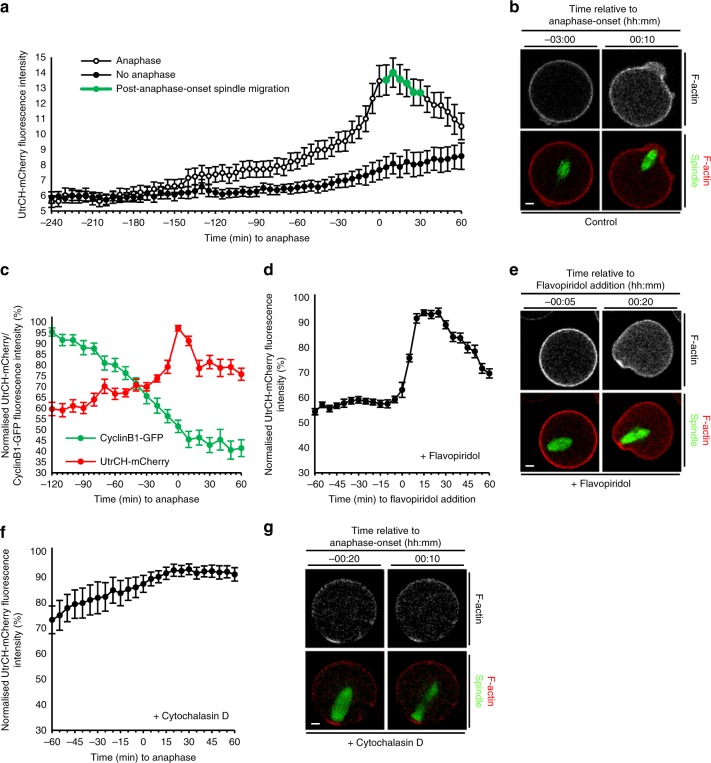
Cdk1 inactivation induces increased F-actin required for spindle migration. **a** Cytoplasmic UtrCH-mCherry intensity in live oocytes relative to anaphase-onset. Shown is a representative replicate in which absolute intensity was determined for oocytes from the same experiment exposed to identical imaging parameters that either underwent anaphase (*n* = 24) or not (no anaphase; *n* = 11). Note that spindle migration (green markers) occurs coincidently with the F-actin spike. **b** Increased cytoplasmic F-actin coincident with anaphase-onset (see Supplementary Movie 21). **c** UtrCH-mCherry and Cyclin B1-GFP fluorescence relative to anaphase-onset in oocytes co-expressing both constructs (*n* = 11). **d**, **e** UtrCH-mCherry fluorescence changes following the addition of flavopiridol at 4 h post-GVBD (*n* = 15; see Supplementary Movie 22). **f**, **g** UtrCH-mCherry fluorescence following cytochalasin D treatment from 6 h post-GVBD (*n* = 11). Note the absence of migration and protrusion following anaphase-onset (see Supplementary Movie 23). Times in panels are hours:minutes relative to anaphase-onset (**b**, **g**) and to flavopiridol addition (**e**), respectively. Scale bars, 10 µm. All error bars are mean ± SEM

We quantified F-actin intensity within the cytoplasmic region adjacent to the posterior pole during exit from MI, incorporating the ~2–3-h period during which cyclin B1 degradation and hence Cdk1 inactivation occurs (see Supplementary Fig. 6). We compared F-actin profiles in oocytes undergoing anaphase (reflecting intact Cdk1 inactivation) with profiles from oocytes that were simultaneously imaged under identical conditions in the same experiment but remained arrested at metaphase (reflecting compromised Cdk1 inactivation). In oocytes that did not undergo anaphase, F-actin levels were largely unchanged until ~1 h prior to the average time of anaphase-onset when they exhibited a slow increase (Fig. 4a). In contrast, when anaphase occurred, we observed two patterns of cytoplasmic F-actin increase. The first was a slow increase of ~28.5% from 2 h pre-anaphase-onset to 30 min pre-anaphase-onset (0.3% per min) coincident with the period when cyclin B1 destruction was underway, suggesting that it could be linked with Cdk1 inactivation. Strikingly, this slow increase changed to a steep increase ~20–30 min prior to anaphase-onset (coinciding with the period when Cdk1 activity is approaching its lowest levels) resulting in an ~47.3% increase in F-actin from 30 min pre-anaphase-onset to 10 min post-anaphase-onset (1.2% per min; Fig. 4a, b and Supplementary Movie 21). Note that the increase in cytoplasmic F-actin is more apparent when the UtrCH-Cherry signal is represented in monochrome rather than as a red pseudo-colour. Significantly, the F-actin spike coincided with the period of spindle migration and protrusion (Fig. 4a, b). To more closely investigate the relationship between F-actin and cyclin B1, we simultaneously imaged in the same oocyte cyclin B1-GFP with UtrCH-mCherry and fluorescent tubulin. We found that anaphase initiated ~10 min prior to the nadir of cyclin B1 (Fig. 4c) confirming that anaphase initiation coincided with almost complete Cdk1 inactivation. Consistent with our previous findings, F-actin levels began increasing steeply during the final 20–30 min of cyclin B1 destruction, with F-actin levels peaking 10 min before cyclin B1 levels were at their lowest (Fig. 4c). Thus F-actin levels undergo a biphasic increase contemporaneously with Cdk1 inactivation, including a steep and transient spike that coincides with near-complete Cdk1 inactivation, anaphase-onset and spindle migration.

To directly test whether inactivation of Cdk1 could induce F-actin polymerisation, we inactivated Cdk1 at a time when its activity is normally high by adding flavopiridol at 4 h post-GVBD, prior to the onset of cyclin B1 destruction (see Supplementary Fig. 6). By introducing flavopiridol while live-cell imaging was underway, the small but distinct oocyte displacement caused when the drug was pipetted into the media served as a visual cue for the precise time that Cdk1 inactivation commenced (see Supplementary Movie 22, Flavopiridol addition frame). Significantly, F-actin intensity increased steeply within 5 min of drug addition and peaked by 15 min (Fig. 4d, e and Supplementary Movie 22). If increased F-actin polymerisation generates a force to push the spindle, then preventing actin polymerisation should prevent spindle migration and protrusion. To test this, we added cytochalasin D^2^ to disrupt the actin meshwork at 6 h post-GVBD, after spindles had migrated to the cortex. We found that the F-actin spike previously associated with anaphase-onset was completely abolished by cytochalasin (Fig. 4f and Supplementary Movie 23). Highly significantly, these anaphase spindles did not migrate and no protrusions formed (29 of 29 oocytes; Fig. 4g and Supplementary Movie 23). Thus Cdk1 inactivation induces F-actin polymerisation that is critical for spindle displacement and protrusion.

### Formins mediate anaphase-onset F-actin polymerisation

In mouse oocytes, formins and Arp2/3 predominantly nucleate cytoplasmic and cortical actin, respectively^3,5,23^. To determine which actin nucleator mediated the F-actin spike, we employed either the formin-specific inhibitor, SMI-FH2^33,34^, or the Arp2/3-specific inhibitor, CK666^23,35^, late in MI after pre-anaphase spindle migration was complete.

**Fig. 5:**
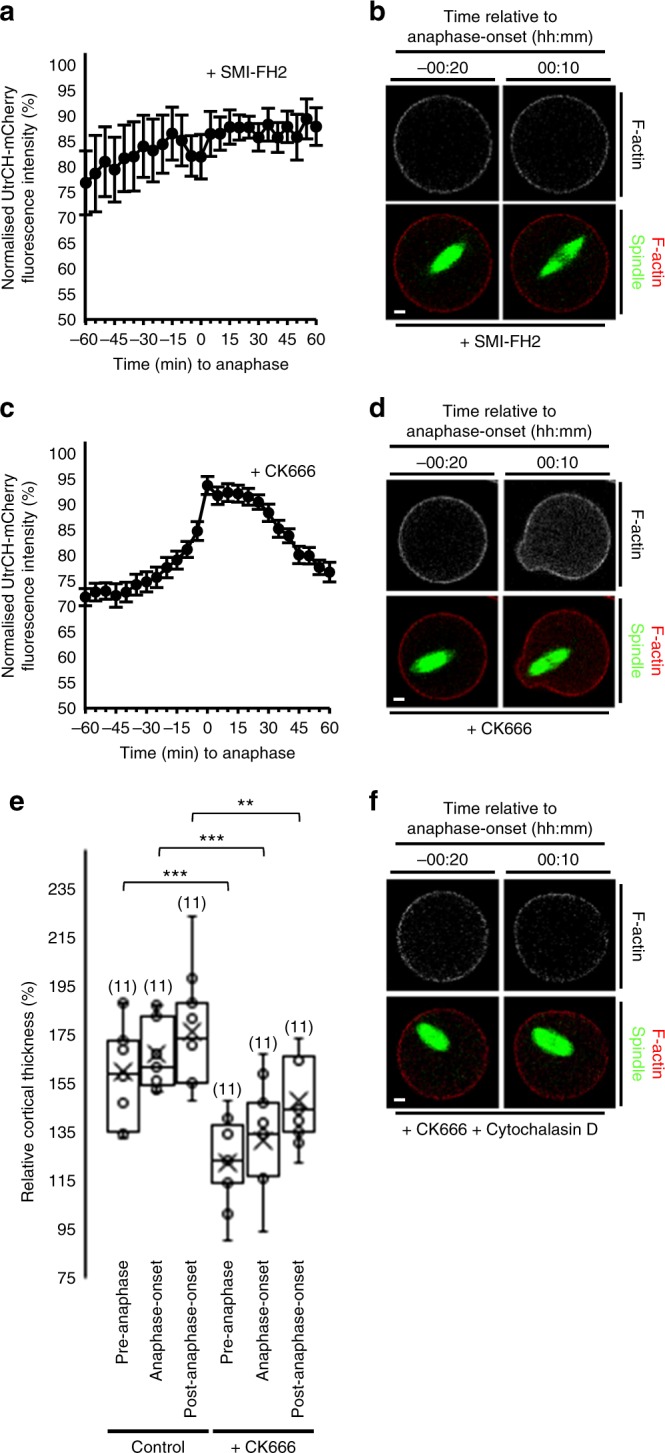
Increased cytoplasmic F-actin is required for spindle migration. **a**, **b** UtrCH-mCherry fluorescence changes following treatment with SMI-FH2 from 6 h post-GVBD (*n* = 10). Note the absence of migration and protrusion following anaphase-onset. **c**, **d** Cytoplasmic UtrCH-mCherry fluorescence changes in relation to anaphase-onset following CK666 treatment from 6 h post-GVBD (*n* = 19; see Supplementary Movie 24). Error bars are mean ± SEM (**a**, **c**). **e** Cortical UtrCH-mCherry fluorescence intensity within the protrusion in relation to anaphase-onset for control and CK666-treated oocytes. Box plots depict median (horizontal line), mean (crosses), 25th and 75th percentiles (boxes) and 5th and 95th percentiles (whiskers). One-way ANOVA used for statistical analysis. *P* values represented as **P < 0.01 and ***P < 0.001. **f** Lack of anaphase-onset-related increase in UtrCH-mCherry fluorescence following co-treatment with CK666+cytochalasin D from 6 h post-GVBD. Times in panels are hours:minutes relative to anaphase-onset. Scale bars, 10 µm. Oocyte numbers are shown in parentheses from a minimum of three independent experiments

We found that treatment with SMI-FH2 prevented the spike in cytoplasmic F-actin (Fig. 5a, b) normally associated with anaphase-onset (see Fig. 4a), whereas CK666 did not (Fig. 5c, d). CK666 did, however, blunt cortical thickening following anaphase-onset (Fig. 5e), consistent with Arp2/3’s known role in modulating cortical F-actin. Significantly, post-anaphase-onset spindle migration and protrusion occurred in 86% of CK666-treated oocytes (30 of 35 oocytes; Fig. 5d and Supplementary Movie 24) compared with only 33% of SMI-FH2-treated oocytes (5 of 15 oocytes; Fig. 5b). Cytoplasmic actin polymerisation in CK666-treated oocytes was important for spindle migration and protrusion since both were abrogated in oocytes co-treated with CK666 and cytochalasin D (22 of 22 oocytes; Fig. 5f).

Collectively, these data show that Cdk1 inactivation triggers marked formin-mediated cytoplasmic F-actin polymerisation that is critical for spindle migration and protrusion. Since protrusion is reliant on changes initiated within the cytoplasm and is unperturbed by disrupting changes in cortical F-actin, these data further support that protrusion is not an independent cortical event.

### Protrusion maximises asymmetry

We reasoned that the shift in midzone position of ~8 µm brought about by anaphase spindle migration (see Fig. 1i) could play a hitherto unrecognised role in influencing asymmetry by displacing the midzone-directed site of furrowing. We imaged the spindle and the cortex and found that acute furrowing occurred at the base of the protrusion, midway along the length of the spindle (Supplementary Fig. 9 and Supplementary Movie 25), confirming that the midzone at the end of anaphase spindle migration defines the site of furrowing.

Since post-anaphase-onset spindle migration determines midzone position and hence the site of furrowing, the extent of migration could be critical for determining the degree of asymmetry. To better interrogate the effects of post-anaphase-onset migration, we sought to exaggerate the distance available for this phase of migration by inhibiting pre-anaphase migration. Our previous data indicated that, to leave post-anaphase-onset migration intact, we needed to target pathways that would not disrupt cytoplasmic F-actin.

**Fig. 6:**
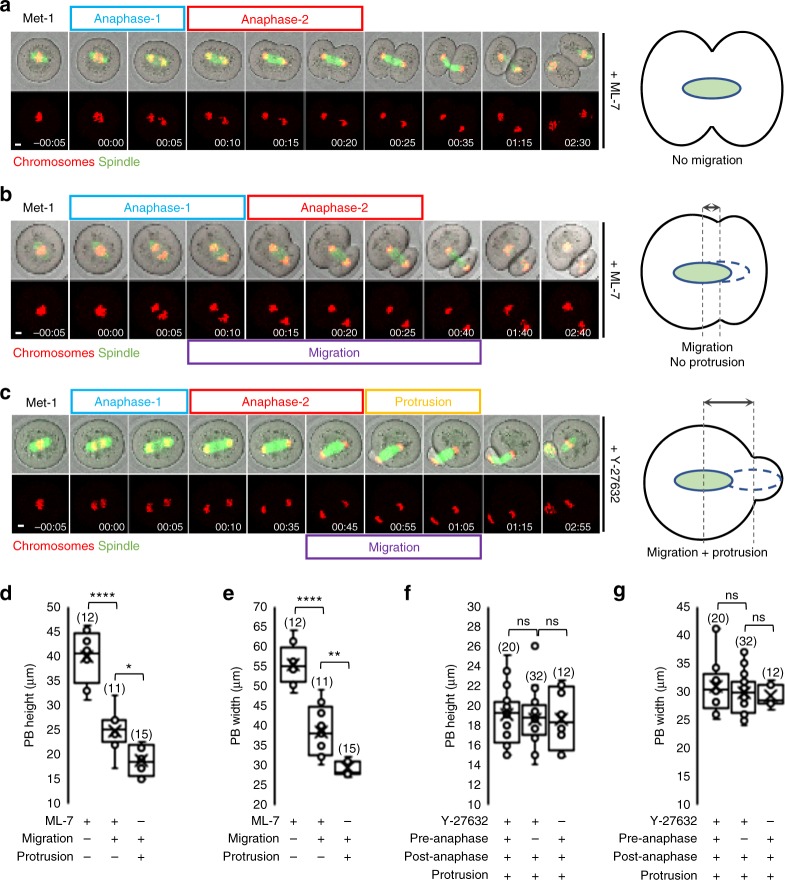
Extreme asymmetry requires anaphase-spindle migration and protrusion. **a**–**c** Live oocytes treated with either ML-7 (**a**, **b**; see Supplementary Movie 26 and 27) or Y-27632 (**c**; see Supplementary Movie 28) undergoing anaphase at the oocyte centre followed by either no spindle migration (**a**; see Supplementary Movie 26), migration to the cortex without protrusion (**b**; see Supplementary Movie 27) or migration and protrusion (**c**; see Supplementary Movie 28) as depicted in schematics. **d**–**g** Polar body (PB) dimensions in ML-7 (**d**, **e**) and Y-27632 (**f**, **g**) treated oocytes. Note that Pre-anaphase and Post-anaphase (**f** and **g**) refer to pre-anaphase and post-anaphase spindle migration, respectively. Box plots depict median (horizontal line), mean (crosses), 25th and 75th percentiles (boxes) and 5th and 95th percentiles (whiskers). One-way ANOVA used for statistical analysis. *P* values represented as *P < 0.05, **P < 0.01, and ****P < 0.0001, ns denoted *P* > 0.05. Times in panels are hours:minutes relative to anaphase-onset. Scale bars, 10 µm. Oocyte numbers are shown in parentheses from a minimum of three independent experiments

To do this, we inhibited myosin II using two small molecule inhibitors, ML-7 and Y-27632, which impaired pre-anaphase spindle migration (Supplementary Fig. 10a) consistent with previous results^4,36^. Remarkably, timelapse imaging revealed that, after undergoing anaphase at the oocyte-centre following inhibitor treatment, 17 of the 27 ML-7-treated and 43 of the 64 Y-27632-treated spindles then migrated towards the cortex to varying extents before furrowing (Supplementary Fig. 10b, c). As expected, for spindles that completed anaphase at the oocyte centre without migrating, near-symmetrical cleavage occurred (Fig. 6a and Supplementary Movie 26). In stark contrast, migration after anaphase-onset displaced the midzone peripherally and resulted in significantly smaller PBs (Fig. 6b, d, e and Supplementary Movie 27). Strikingly, many spindles (predominantly following Y-27632 treatment, 38 of 43 oocytes) not only migrated after anaphase-onset all the way to the nearest cortex but they then also induced a protrusion (Fig. 6c and Supplementary Movie 28). Throughout their extended period of post-anaphase spindle migration, Y-27632-treated oocytes never exhibited a protrusion before the spindle reached the cortex, reinforcing the dependence of protrusion on spindle migration into the cortex. Notably, and in keeping with the critical importance of cytoplasmic F-actin polymerisation for post-anaphase migration, we found that cytoplasmic F-actin levels increased markedly following anaphase-onset in Y-27632-treated oocytes (Supplementary Fig. 11a, b). Moreover, preventing this increase by co-treating Y-27632 oocytes with cytochalasin D completely prevented post-anaphase spindle migration and protrusion (Supplementary Fig. 11c, d).

Highly significantly, PB size when spindles migrated post-anaphase-onset and then produced a protrusion (migration and protrusion) were identical to untreated controls (Fig. 6c, f, g). In contrast, PBs were larger than controls if post-anaphase spindle migration, but no protrusion (migration-only), occurred (Fig. 6b, d, e). However, the latter migration-only PBs were smaller than if no migration occurred (Fig. 6a, d, e). Thus post-anaphase-onset spindle migration strongly biases asymmetry that is maximised when accompanied by protrusion. In other words, displacement of the spindle towards the cortex is not sufficient on its own for producing the smallest PB. Interestingly, spindle migration following anaphase could completely compensate for failure of pre-anaphase migration for achieving asymmetry (Fig. 6c, f, g and Supplementary Movie 28).

### Mos depletion impairs post-anaphase-onset spindle migration

Because the Mos/MAPK pathway is known to be important for asymmetric division^13,37^, we were curious as to whether it might also impact post-anaphase-onset events in addition to its known role in pre-anaphase migration. To investigate this, we depleted Mos using a previously validated morpholino^37,38^. Consistent with effective knockdown, we found unusually large PBs in 49% of oocytes (53 of 108) and 15% of oocytes extruded a second PB (16 of 108; Supplementary Fig. 12), very similar to previously published rates using this same morpholino sequence^37^. In contrast, enlarged PBs occurred in only 14% (9 of 64) of mock-depleted oocytes with none extruding a second PB (Supplementary Fig. 12).

**Fig. 7:**
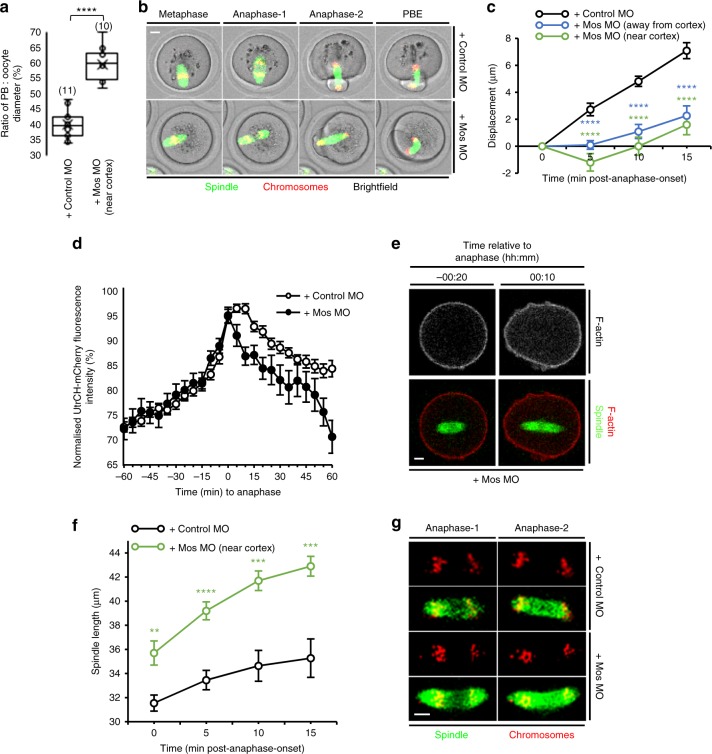
Mos depletion impacts anaphase-spindle migration and spindle length. **a** Ratio of PB:oocyte diameter for mock-depleted oocytes and Mos-depleted oocytes having cortical-proximal spindles at the time of anaphase-onset. Oocyte numbers are shown in parentheses from a minimum of three independent experiments. **b** Large PB produced following Mos depletion despite the spindle being adjacent to the cortex at anaphase-onset (see Supplementary Movie 29). **c** Quantification of spindle displacement (due to the marked anaphase spindle elongation [see **d**], midzone displacement rather than posterior pole displacement was quantified) between anaphase-onset and anaphase-completion for Mos- and mock-depleted oocytes (*n* = 11). For Mos-depleted oocytes, results were grouped based on whether spindles underwent anaphase close to the oocyte centre (MosMO (away from cortex), *n* = 12) or close to the cortex (MosMO (near cortex), *n* = 10). **d** Cytoplasmic UtrCH-mCherry fluorescence in relation to anaphase-onset for mock- and Mos-depleted oocytes (*n* = 20 and *n* = 17, respectively). **e** Live Mos-depleted oocyte in which spindle undergoes anaphase close to the oocyte centre associated with increased cytoplasmic F-actin. **f**, **g** Changes in spindle lengths between anaphase-onset and anaphase-completion for live mock- and Mos-depleted oocytes (*n* = 11 and *n* = 10, respectively). Times in panels are hours:minutes relative to anaphase-onset. Scale bars, 10 µm. Two-tailed Student’s *t* test (**a**, **f**) or one-way ANOVA (**c**) used for statistical analysis. *P* values represented as **P < 0.01, ***P < 0.001 and ****P < 0.0001

We were struck that, in 10 of the 29 oocytes with large PBs, the spindle underwent anaphase close to the cortex, yet large PBs were produced (Fig. 7a, b and Supplementary Movie 29), indicating that asymmetry was compromised despite intact pre-anaphase spindle migration. Unexpectedly, we found that post-anaphase-onset migration was markedly impaired in Mos-depleted oocytes regardless of the position of the spindle relative to the cortex when anaphase initiated (Fig. 7c). We note that cytoplasmic F-actin is unaffected in *MOS*^*−/−*^ oocytes before anaphase-onset^23^, but wondered whether lack of Mos might impact the post-anaphase-onset period. Although an F-actin spike of similar magnitude to control oocytes was observed at anaphase-onset in Mos-depleted oocytes, the duration of the spike was noticeably shorter (Fig. 7d, e). Also, and consistent with earlier data^13^, we found that anaphase-spindle elongation was significantly greater following Mos depletion than in controls (Fig. 7f, g).

Thus our data support that, following Mos depletion, at least two factors might impair spindle migration after anaphase-onset and thereby compromise asymmetry: shorter-lasting F-actin spikes and larger anaphase spindles. We note as well that, by positioning the midzone deep within the oocyte, longer anaphase spindles would predispose to large PBs independently of spindle migration^13^. This genetic approach therefore strongly supports our previous findings using small molecule inhibitors that post-anaphase-onset spindle migration is critical for asymmetry.

### Migration and midzone-to-cortex distance influence furrowing

**Fig. 8:**
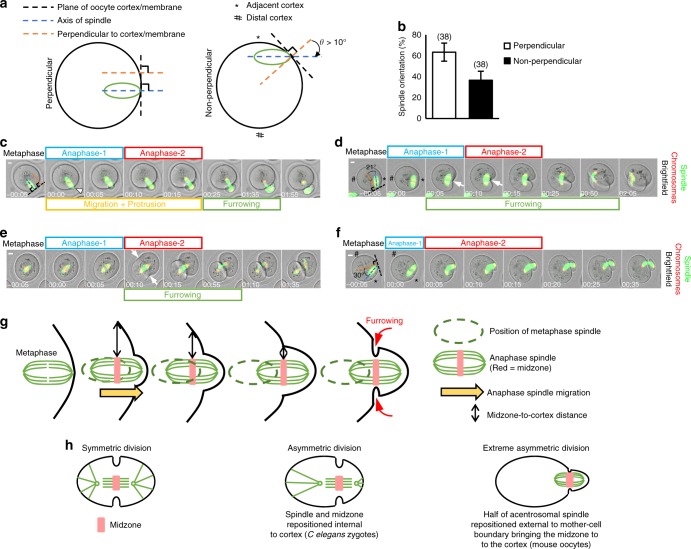
Spindle migration following anaphase-onset delays furrowing. **a** Schematic for defining orientation of cortex-proximal spindles relative to cortex. Spindles were considered non-perpendicular if *θ* > 10°. **b** Spindle orientation at anaphase-onset. Error bars are mean ± SEM. Oocyte numbers are shown in parentheses from a minimum of three independent experiments. **c**–**f** Untreated oocytes, which at the time of anaphase-onset, have spindles located either at the cortex (**c**, **d**, **f**) or at the oocyte-centre (**e**) and oriented either perpendicular (**c**) or non-perpendicular (**d**, **f**). Typically, asymmetry results after perpendicular spindles migrate into the membrane following anaphase-onset leading to displacement of around half of the spindle beyond the oocyte (**c**; see Supplementary Movie 30). Near-symmetrical cleavage if cortically located non-perpendicular spindles do not induce migration/protrusion (**d**; see Supplementary Movie 31). Furrowing (arrowhead) occurs at the midzone-proximal membrane (*) but not at the distal membrane (#) for cortically located spindles (**d**) and is two-sided in the case of central spindles (arrowheads, **e**; see Supplementary Movie 32). Migration/protrusion enables cortically located non-perpendicular spindles to produce extreme asymmetry (**f**; see Supplementary Movie 33). **g**, **h** Model of anaphase-spindle migration/protrusion that positions half of the spindle external to the oocyte (**g**) and comparison with symmetry and asymmetry models in which the anaphase spindle remains internal to the mother cell (**h**). Times in panels are hours:minutes relative to anaphase-onset. Scale bars, 10 µm

Most oocytes initiated anaphase with their spindles oriented perpendicular to the cortex (Fig. 8a, b). In such cases, a midzone was present for at least 15–20 min without acute furrowing, which only occurred when the midzone arrived at the base of the protrusion coincident with anaphase-2 completion (Fig. 8c and Supplementary Movie 30; see also Fig. 1f and Supplementary Fig. 9). In contrast, in the case of non-perpendicular spindles, furrowing occurred around 10 min earlier during anaphase-1 (*n* = 11; Fig. 8d and Supplementary Movie 31); hence, very shortly after formation, the midzone is fully capable of inducing furrowing. How then is furrowing ordinarily delayed in the case of perpendicular spindles until the very end of anaphase-2? One obvious difference was that, for non-perpendicular spindles, the mean midzone-to-cortex distance at anaphase-onset was 7.1 µm (*n* = 14), whereas for perpendicular spindles it was over three-fold greater (23.8 µm; *n* = 16; *P* < 0.0001; Student’s *t* test). This suggested that spindle position in relation to the cortex could influence the strength of the midzone-derived signal reaching the cortex. In line with distance effects, non-perpendicular spindles induced furrowing at the nearby cortex but not at the contralateral (more distal) side when in an off-centre location (*n* = 11; Fig. 8d), whereas two surfaces furrowed almost simultaneously when the spindle was centrally located (*n* = 6; Fig. 8e and Supplementary Movie 32). Therefore, the interval between anaphase-onset and furrowing is dependent upon midzone-to-cortex distance consistent with previous findings during MII^12^. Notably, however, during MI, such distance relationships are strongly influenced by relatively subtle changes in the orientation of the long spindle axis relative to the cortex.

Highly significantly, for non-perpendicular spindles undergoing anaphase at the cortex, division asymmetry was influenced by whether migration with protrusion occurred. If neither pole induced protrusion and the midzone remained stationary relative to the overlying cortex, the furrow then tracked through the oocyte centre resulting in near-symmetrical cleavage (Fig. 8d and Supplementary Movie 31). Notably, an almost identical pattern of cleavage is obtained if the cortex is compressed at the site of the metaphase I spindle thereby immobilising it and preventing migration and protrusion^39^. In striking contrast, if one pole of a non-perpendicular spindle became anchored to the cortex after anaphase-onset, furrowing was delayed in favour of spindle migration and protrusion until the end of anaphase-2, ultimately leading to asymmetric division (*n* = 13; Fig. 8f and Supplementary Movie 33). This strongly suggests that at least two co-operative events, the midzone-to-cortex distance and anaphase spindle migration, reduce the ability of the midzone to sufficiently modify a focal region within the overlying cortex thereby disfavouring furrowing until the midzone arrives at the base of the protrusion where the midzone-to-cortex distance is minimal (Fig. 8g).

## Discussion

Here we identify a phase of spindle displacement that occurs post-anaphase-onset secondary to Cdk1 inactivation. Reduced Cdk1 activity also induces spindle displacement in *C. elegans* zygotes^40^ supporting a broader role for Cdk1 in spindle motility. In contrast to *C. elegans* zygotes, however, spindle migration in mouse oocytes occurs after anaphase-onset and, importantly, results in protrusion that is critical for maximal asymmetry (Fig. 8h). High Cdk1 activity is known to inhibit separase^26^, explaining the close relationship between Cdk1 inactivation, anaphase-onset and spindle migration.

Since F-actin polymerisation is well known to generate propulsive force^32^, we hypothesise that formin-mediated F-actin polymerisation induced by Cdk1 inactivation generates a force. We propose that this force pushes the anaphase spindle (manifested as anaphase-spindle migration) against the cortex thereby inducing protrusion. In this model, the spindle constitutes a mechanical transmitter as supported by the requirement of an intact spindle for sustained protrusions (Fig. 3). We do not rule out the possibility that short-range signals emanating from the cortex-proximal anaphase spindle could also contribute to protrusion formation. However, our data do not support that such signals, if they exist, are sufficient for protrusion (see Figs. 4g and 5f in which a cortex-proximal spindle undergoes anaphase, but in the absence of the cytoplasmic F-actin spike, no spindle migration and no protrusion occur). We did not measure tension within the protrusion and therefore do not rule out the possibility that cortical weakening could also facilitate protrusion although we note that this is not supported by the F-actin thickening we observed within the protrusion (Supplementary Fig. 7). Additionally, thinning of the protrusion cortex following Arp2/3 inhibition did not appear to predispose to overt PB enlargement (Fig. 5c, d) as might be expected if reduced cortical tension was critical for forming the protrusion. The transient nature of the F-actin spike is likely important for limiting force duration and, consequently, protrusion size. Conversely, as our data from Mos-depleted oocytes suggest, too short a spike could potentially compromise migration and hence protrusion. Increased oocyte force is also triggered concurrently with anaphase I in *C. elegans* oocytes, although in this case, force is myosin II mediated^41^. In *C. elegans* oocytes, force generation requires separase activation^41^, which is also likely important in mouse oocytes since PBE is impaired in separase-knockout oocytes^7^ and separase is required for inactivating Cdk1^42^.

Employing the same cell-cycle cue (Cdk1 inactivation) for inducing both anaphase and protrusion ensures that these two exit events remain tightly coupled. Notably, in mouse oocytes, Cdk1 inactivation might also activate the Rho-GEF, Ect2, and downstream RhoA-mediated furrowing^16^. Cdk1 inactivation could therefore underpin key events required for MI exit. If triggered by a common cue, how would these events be constrained to occur in the correct temporal sequence? Our data support that at least one mechanism for enforcing a delay between anaphase-onset and furrowing is spindle migration. In *C. elegans* oocytes, the leading spindle pole remains stationary and the cortex is pulled down over the spindle^41^. Although distinctly different from spindle migration, the net effect in both cases is relative movement between the midzone and overlying cortex, pointing to common underlying events for delaying furrowing. Proper ordering of events during MI exit could also involve event-specific Cdk1 activity thresholds, akin to the model proposed for mitotic exit^43^. Since anaphase begins around 10 min before cyclin B1 levels are at their lowest, it is possible that each subsequent event is triggered as lower Cdk1 activity thresholds are reached during this terminal window of Cdk1 inactivation. Late furrowing could also involve the chromosomal passenger complex (CPC) since oocytes in which the catalytic CPC components, Aurora kinases B and C, are disrupted using a dominant-negative construct undergo protrusion but furrowing fails^44,45^.

Exactly which Cdk1 substrate(s) undergoes dephosphorylation to directly or indirectly augment F-actin polymerisation is not currently known but several components of actin-related pathways are Cdk1 substrates^46^. Interestingly, the activity of the fission yeast formin, Cdc12, is modulated by Cdk1-dependent phosphorylation^47^. It is significant in this regard that our results support that formins mediate F-actin polymerisation post-Cdk1 inactivation. Intriguingly, Formin2 expression increases during late MI in mouse oocytes and peaks around the time of anaphase-onset^48^ raising the possibility that high formin levels could prime the peri-anaphase actin surge. Formin2-mediated actin polymerisation also promotes spindle displacement during early MI^49^. This raises the possibility that formin-dependent spindle mobilisation occurs during periods of relatively low Cdk1 activity (early MI and post-anaphase-onset). Conversely, Arp2/3 could predominate in high-Cdk1-activity environments, explaining the importance of Arp2/3-mediated cytoplasmic flow during late pre-anaphase spindle migration^49^ and in MII^50^ but not in the intervening low-Cdk1-activity period (our data).

Our findings in Mos-depleted oocytes suggest that the other major oocyte kinase and target of Mos, MAPK, is also important for post-anaphase-onset migration, in part by influencing the duration of the F-actin spike as well as indirectly by restraining spindle growth during anaphase. Given that MAPK modulates APC-mediated cyclin B1 destruction in late MI^38^, it is possible that Mos depletion impacts MAPK-dependent control of Cdk1 inactivation and hence F-actin polymerisation.

In closing, we note that the GV, and hence spindle, often assumes an off-centre position in follicle-enclosed mouse oocytes, suggesting that surrounding somatic cells contribute to asymmetry^51^. Since our experiments required denuded oocytes for detailed imaging, we cannot exclude that additional somatic inputs also contribute to protrusion formation in follicle-enclosed oocytes in vivo.

## Methods

### Oocyte isolation, culture and microinjection

All animals were housed in a specific pathogen-free environment in filter-top cages and fed a standard diet. All work involving animals complied with all relevant ethical regulations and was approved by the Animal Ethics Committee at the University of Queensland. Oocytes were isolated from the ovaries of 3–4-week-old B6CBAF1 female mice 44–46 h following intra-peritoneal injection of 7.5 international units (IU) of pregnant mare’s serum gonadotrophin (Pacificvet). Dissected ovaries were immediately transferred to the laboratory in pre-warmed αMEM HEPES-buffered medium (Sigma-Aldrich) containing 50 μM 3-isobutyl-1-methylxanthine (IBMX; Sigma-Aldrich), which prevents oocytes from undergoing GVBD^52,53^. Ovarian antral follicles were punctured in IBMX-treated media in 35 × 10 mm^2^ dishes using a 27 G needle under direct vision on the stage of a stereomicroscope (M165C, Leica Microsystems). Only fully grown cumulus-covered oocytes were isolated and used for further experiments. For longer-term culture and for all confocal imaging, oocytes were cultured in micro-drops of M16 media (Sigma-Aldrich) under embryo-tested light mineral oil (Sigma-Aldrich) at 37 °C in an atmosphere of 5% CO_2_ in air^52–54^.

For undertaking microinjection^52–54^, GV-stage oocytes in IBMX-treated medium were stabilised using suction applied through a hydraulic syringe to a pre-fabricated holding pipette (inner diameter 15 μm, outer diameter 75 μm, 35° bend; The Pipette Company). Microinjection needles were pulled from capillary tubes (0.86 mm inner diameter, 1.5 mm outer diameter; Harvard Apparatus) to a pre-determined calibre using a vertical pipette puller (P30 vertical micropipette puller, Sutter Instruments). The tip of the microinjection pipette was advanced across the zona pellucida and oolemma into the cytoplasm of the oocyte aided by a brief electrical pulse delivered by an intracellular electrometer (IE-25IA, Warner Instruments). A precise volume roughly equal to 5% of the oocyte volume of test solution was then delivered to the oocyte using a Pneumatic PicoPump (PV-820, World Precision Instruments). The rate of oocyte death following microinjection was consistently <10%.

### cRNA constructs and morpholinos

The mMESSAGE mMACHINE High Yield Capped RNA Transcription Kit (Ambion) was used to produce cRNA constructs by T3-promoter driven in vitro transcription from linearised DNA template^53,54^. Constructs used in this paper were histone 2B (H2B)-RFP, Securin-GFP, Cyclin B1-mCherry, UtrCH-mCherry (a gift from William Bement; Addgene plasmid # 26740^22^) and MAP7-GFP cRNAs. All plasmids were fully sequenced prior to transcription. Following in vitro transcription, cRNA size was verified on agarose gels and concentrations were determined using a spectrophotometer. Constructs were microinjected at the following concentrations: H2B-RFP at 250 ng/μl, Securin-GFP at 200 ng/μl, Cyclin B1-mCherry at 200 ng/μl, UtrCH-mCherry at 650 ng/μl, and MAP7-GFP at 700 ng/µl. Following microinjection at the GV stage, oocytes were held in 50 μM IBMX for at least 2 h to allow time for protein translation while maintaining GV arrest. Oocytes were then washed through 5–6 drops of IBMX-free media to allow resumption of maturation.

For depleting Mos, GV-stage oocytes were microinjected with a previously published morpholino sequence designated Mos MO that was designed to target *MOS* (NM_020021.2 [https://www.ncbi.nlm.nih.gov/nuccore/NM_020021]; 5′–CACAGGCTTAGAGGCGAAGGCATT–3′) (GeneTools)^37,38^. Following microinjection, oocytes were maintained in IBMX-treated medium for 24 h to allow time for protein knockdown. For mock depletion, GV-stage oocytes were microinjected with a standard control morpholino (designated Control MO)^53,54^. Morpholinos were injected at a concentration of 3 mM.

### Drug additions

Stock solutions of all small molecule inhibitors were made in DMSO at the highest possible concentration that enabled complete solubilisation thereby minimising the volume of DMSO added to media. For inhibiting Cdk1 activity, flavopiridol (SelleckChem; 10 mM stock solution) was dissolved in media for a final concentration of 5 μM as used previously in mammalian somatic cells^27^ and mouse oocytes^28,29^. RO-3306^31^ (Sigma-Aldrich; 20 mM stock solution) was also used to inhibit Cdk1 activity at 10 μM. Either the APC-Cdc20-specific inhibitor, APCIN (Sigma-Aldrich; 50 mM stock solution)^30^, or the 26S proteasome inhibitor, MG132 (SelleckChem; 10 mM Stock solution), were used to inhibit proteolysis at 4 h post-GVBD before proteolysis has begun (Supplementary Fig. 6) at 150 and 5 μM, respectively. To inhibit MAPK activity late in meiosis, at 6 h post-GVBD, similarly to the Cdk1 inhibition described earlier, U0126 (Sigma-Aldrich; 20 mM stock solution) was used at a concentration of 50 μM. Myosin II activity was inhibited prior to spindle migration at 2 h post-GVBD (Supplementary Fig. 1) by addition of either Y-27632^16,36^ (50 μM) (Sigma-Aldrich; stock solution 10 mM) or ML-7^4,23^ (30 μM) (Sigma-Aldrich; stock solution 30 mM). Nocodazole was used to depolymerise the fully assembled spindle at 6 h post-GVBD at a concentration of 10 μM (Sigma-Aldrich; stock solution 10 mM) for 20 min. Actin polymerisation was disrupted at 6 h post-GVBD using cytochalasin D at 1 µM (Sigma-Aldrich; stock solution 2 mM), SMI-FH2 at 25 µM (Sigma-Aldrich; stock solution 50 mM) or CK666 at 200 µM^23^ (Sapphire Bioscience; stock solution 50 mM). As a control, DMSO, which was used to dissolve all inhibitors, was added to media at a final concentration of 0.2%, very similar to the concentration attained when inhibitors were added.

### Timelapse confocal microscopy

Timelapse microscopy was used to simultaneously image combinations of spindles, chromosomes and membrane behaviour in live mouse oocytes or spindles and cortical F-actin at high temporal resolution, specifically during the narrow window between anaphase-onset and completion of PB formation (marking completion of first meiotic cytokinesis). To visualise microtubules, SiR-Tubulin dye (Cytoskeleton, Inc.) was added to media at a final concentration of 100 nM. We favoured the SiR-Tubulin dye for imaging spindles since it allowed for excitation in the far-red channel. Moreover, in addition to enabling use of the low-toxicity far-red laser, the configuration of our Leica SP8 TCS microscope, which included Leica’s HyD ultra-sensitive detection system, enabled very low laser power (typically <2%, see below) to be used, thereby limiting photo-damage to the live oocytes that could compromise maturation competence. At the low concentrations used (dilution factor of 1:10,000), SiR-Tubulin has been found to have no adverse effects on cell-cycle progression and no cytotoxic effects^55^. This is supported by recent work in mouse oocytes, which validated this dye at this concentration for imaging spindles^19,20^.

For simultaneously imaging spindles, chromosomes and membranes, or F-actin and spindles, fluorescence images of spindles and chromosomes as well as brightfield images of oocytes were acquired using a Leica TCS SP8 confocal microscope equipped with a 20×/0.75 NA Apochromat water-immersion objective fitted with an automated pump cap (water immersion micro dispenser, Leica; automated pump mp-x controller, Bartels Mikrotechnik). This pump delivered water from a reservoir to the cap at a pre-programmed rate, thereby counteracting evaporation within the heated microscope incubator to ensure continuous water coverage of the objective during extended experiments.

Oocytes were imaged in 1–2 μl micro-drops of M16 medium in glass-bottom dishes (35 × 10 mm^2^ dish, no. 0 coverslip; MatTek) with or without inhibitors, under mineral oil. For the entire duration of imaging, oocytes were enclosed within a purpose-built stage-mounted incubation chamber designed to maintain conditions of 37 °C and 5% CO_2_ in air. Temperature fluctuation was further buffered by enclosing the entire microscope, including the stage-mounted chamber, in a custom-designed polycarbonate incubator (Life Imaging Services) that maintained a stable internal temperature of 37 °C. Automated image capture was driven by the Leica LAS X software. The Leica TCS SP8 microscope was equipped with a Super Z Galvo stage, which allowed ultra-rapid movement in the *z*-plane with extreme precision. Acquiring each *z*-stack at each position was typically completed in <10 s. During long-term imaging, the adaptive focus control facility used an infrared beam to correct for focal drift brought about by thermal-induced fluctuations in the distance between the specimen and objective. At the commencement of imaging, the positions of the spindles in the *z* axis were located. The complete stack was then derived by setting the upper and lower limits at 25 μm above and below this plane, creating a stack of 50 μm. *Z*-stacks were acquired with step intervals of 3.5 μm at 5 min intervals at a speed of 600 Hz. Using the Leica mark-and-find tool, we typically imaged multiple groups of oocytes in separate droplets in each experiment. Leica HyD detectors enabled the highly sensitive detection of very low levels of fluorophore emission, ultimately minimising the required laser excitation levels. The 561 and 633 nm laser lines were used at <0.2% and <2% power, respectively. For the majority of experiments, we minimised laser energy exposure by imaging in the red and far-red end of the spectrum.

Post-acquisition image processing was performed using the Leica LAS X software and images were assembled into panels using Adobe Photoshop. Fluorescence images and movies were produced by merging a maximum projection of the fluorescent channels with the brightfield channel. Fluorescence images and movies showing F-actin and spindles were produced by merging a maximum projection of the spindle channel and a single plane of F-actin.

### Quantification of spindle dimensions and cortical dynamics

For analysis of spindle size and migration, only oocytes having bipolar spindles that were oriented in the horizontal plane and that remained strictly in the same orientation throughout anaphase, protrusion and cytokinesis were included in analyses.

Spindle dimensions were measured only from spindles that remained in the horizontal plane throughout anaphase (see Fig. 1f). Spindle length was measured manually as the pole-to-pole distance along the long axis of the spindle. Spindle midzone dimensions were measured manually, as the maximal distance between the extreme limits of the spindle along a line positioned roughly midway between the two groups of segregating chromosomes and perpendicular to the long axis of the spindle.

During spindle migration, the leading pole was considered as the pole that migrated into the anterior oocyte membrane following anaphase-onset. The other (lagging) pole was the posterior pole, which was closest to the posterior oocyte membrane (see Fig. 1e). To quantify migration, the distance between the posterior pole and the posterior oocyte membrane (posterior pole-to-membrane distance), was determined at successive time points throughout anaphase. Migration distance was then derived from the posterior pole displacement, which was the difference between the posterior pole-to-membrane distance at one time point and that at subsequent time points (*y* − *x* in Fig. 1e).

The term protrusion was used to refer to the outpocketing that occurred following anaphase-onset, partially enveloping one-half of the spindle and extended above the level of the surrounding oocyte membrane. Protrusion height was determined as the distance from the base of the protrusion (at the level of the surrounding oocyte membrane) to the most extreme aspect of the outpocketing (determined from either the membrane or the cortical F-actin signal) along the line of the long axis of the spindle (Fig. 1e).

Spindles were categorised as perpendicular or non-perpendicular using the criteria shown in Fig. 8a. The plane of the membrane/cortex was determined as the tangent to the point at which the leading spindle pole encroached the membrane/cortex in the frame immediately preceding anaphase-onset. The longitudinal axis of the spindle was determined by tracing a line along the long axis of the metaphase spindle in the frame immediately preceding anaphase-onset. The angle, *θ*, was the angle between the longitudinal axis of the spindle and the perpendicular to the plane of the membrane/cortex (see Fig. 8a). Spindles were considered perpendicular if *θ* < 10° (see Fig. 8c) and non-perpendicular for *θ* ≥ 10° (see Fig. 8d, f).

### Quantification of cytoplasmic F-actin

UtrCH-mCherry fluorescence intensity was used for quantifying F-actin levels in live oocytes^22,23^. Cytoplasmic UtrCH-mCherry intensity was determined by measuring the mean background-corrected fluorescence intensity within a cortex-free region drawn immediately adjacent to the lagging spindle pole. To analyse F-actin levels in anaphase and non-anaphase oocytes in Fig. 4a, absolute fluorescence intensity values were plotted over time since the true scale of the difference between the two groups would be lost if normalised values were used. Because oocytes from both groups were analysed within the same experiment, they were microinjected with near-identical cRNA quantities and imaged using identical exposure conditions, allowing us to use changes in absolute fluorescence intensities as a reliable means for comparing within-group changes over time as well as for comparing between-group differences. For all other plots of UtrCH-mCherry fluorescence, intensity was normalised to the maximum value for each individual oocyte.

### Statistical analysis

GraphPad Prism was used to calculate mean and standard error of the mean (SEM). Statistical comparisons were made using either a two-tailed Student’s *t* test with Welch’s correction (for comparing two experimental groups) or one-way analysis of variance with Tukey’s multiple comparisons post-test (for comparing three or more experimental groups) as appropriate to the data set. Graphs were prepared in Microsoft Excel. Box plots were used to depict median (horizontal line), mean (crosses), 25th and 75th percentiles (boxes) and 5th and 95th percentiles (whiskers). *P* values were represented as *P < 0.05, **P < 0.01, ***P < 0.001 and ****P < 0.0001, ns denoted *P* > 0.05. In Figures, oocyte numbers are shown within parenthesis. All experiments were repeated a minimum of three times.

## Electronic supplementary material

Supplementary Information

Description of Additional Supplementary Files

Supplementary Movie 1

Supplementary Movie 2

Supplementary Movie 3

Supplementary Movie 4

Supplementary Movie 5

Supplementary Movie 6

Supplementary Movie 7

Supplementary Movie 8

Supplementary Movie 9

Supplementary Movie 10

Supplementary Movie 11

Supplementary Movie 12

Supplementary Movie 13

Supplementary Movie 14

Supplementary Movie 15

Supplementary Movie 16

Supplementary Movie 17

Supplementary Movie 18

Supplementary Movie 19

Supplementary Movie 20

Supplementary Movie 21

Supplementary Movie 22

Supplementary Movie 23

Supplementary Movie 24

Supplementary Movie 25

Supplementary Movie 26

Supplementary Movie 27

Supplementary Movie 28

Supplementary Movie 29

Supplementary Movie 30

Supplementary Movie 31

Supplementary Movie 32

Supplementary Movie 33

## Data Availability

The data that support the findings of this study are available from the corresponding author upon reasonable request.
