# Supplementary material for: Cdk1 inactivation induces post-anaphase-onset spindle migration and membrane protrusion required for extreme asymmetry in mouse oocytes
Source: Nat Commun. 2018 Oct 2;9:4029. doi: 10.1038/s41467-018-06510-9 (PMC6168559; doi:10.1038/s41467-018-06510-9)
Supplement: Supplementary file 1 — Supplementary Information [file 41467_2018_6510_MOESM1_ESM.pdf]

Supplementary Information

Cdk1-inactivation induces post-anaphase-onset spindle migration and membrane protrusion required for extreme asymmetry in mouse oocytes

Wei et al.

Title: Cdk1-inactivation induces post-anaphase-onset spindle migration and membrane protrusion required for extreme asymmetry in mouse oocytes

Authors: Zhe Wei[1, 2, †], Jessica Greaney[1, 2, †], Chenxi Zhou[1, 2] and Hayden Homer[1, 2, *]

[†]Equal contribution.

Affiliation:

[1]The Christopher Chen Oocyte Biology Research Laboratory, Centre for Clinical Research, Faculty of Medicine, The University of Queensland, Herston, QLD 4029, Australia.

[2]School of Biomedical Sciences, The University of Queensland, St Lucia, QLD 4072, Australia.

***Corresponding Author:** Professor Hayden Homer

*Correspondence to: h.homer@uq.edu.au

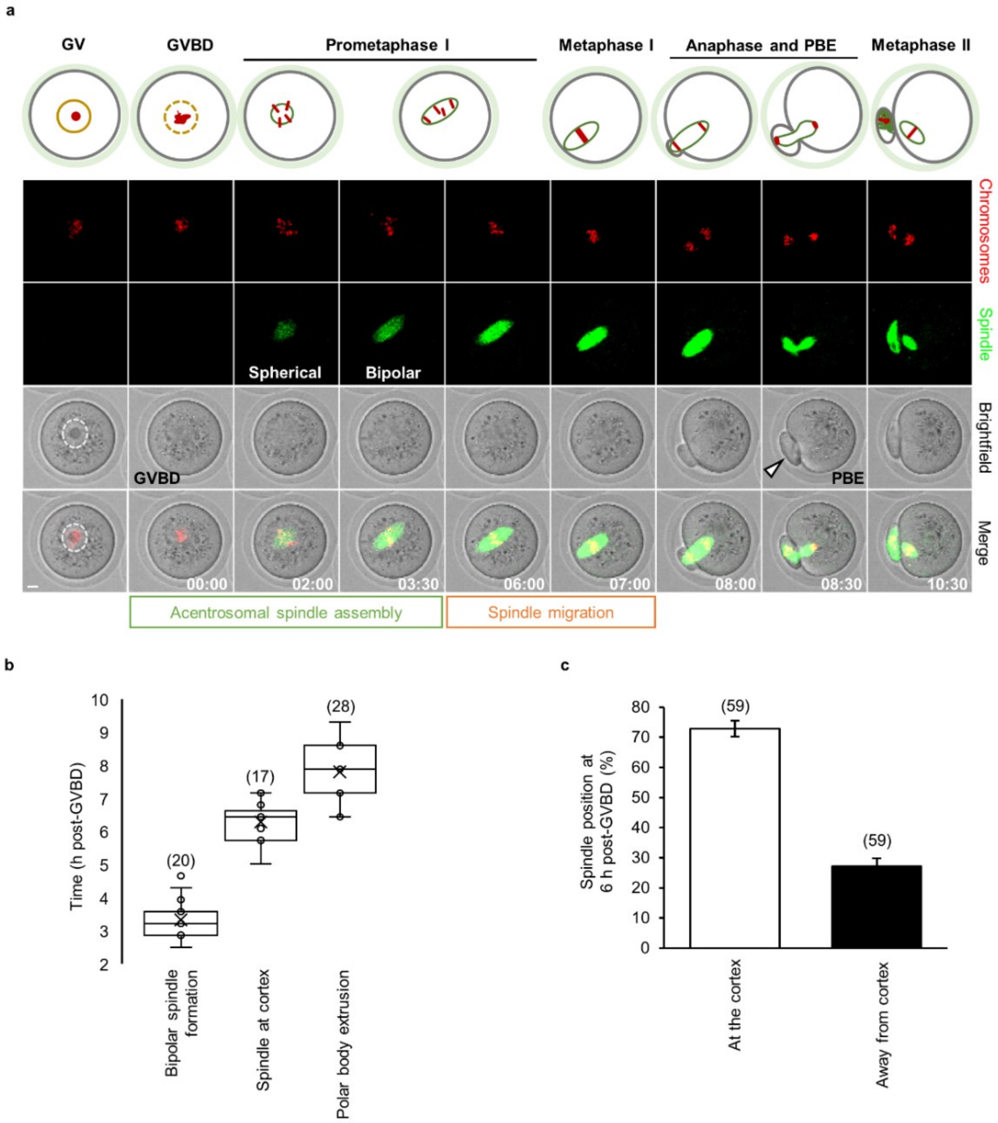

Supplementary Figure 1.

Maturation, acentrosomal spindle assembly and pre-anaphase spindle migration. (**a**) Shown is a schematic of meiotic maturation (top) and a panel comprised of selected frames of fluorescence and brightfield images from a representative timelapse series (below). Times are shown as hours:minutes relative to GVBD. Scale bar, 10 µm. Note that the early-stage spindle is roughly spherical and is remodelled into a bipolar spindle, which then migrates to the cortex. Anaphase and first polar body extrusion (PBE) then ensue following which, oocytes immediately enter meiosis II where they arrest at metaphase II. The dashed white circle and white arrowhead highlight the GV and PB, respectively. (**b**) Timing of bipolar spindle formation, arrival of spindles at the cortex and PBE. Box plots depict median (horizontal line), mean (crosses), 25th and 75th percentiles (boxes) and 5th and 95th percentiles (whiskers). (**c**) Proportions of spindles located either at the cortex or close to the oocyte centre at 6 h post-GVBD. Data in graphs are presented as the mean ± SEM. Oocyte numbers are shown in parenthesis from three independent experiments. See also Supplementary Movie 1.

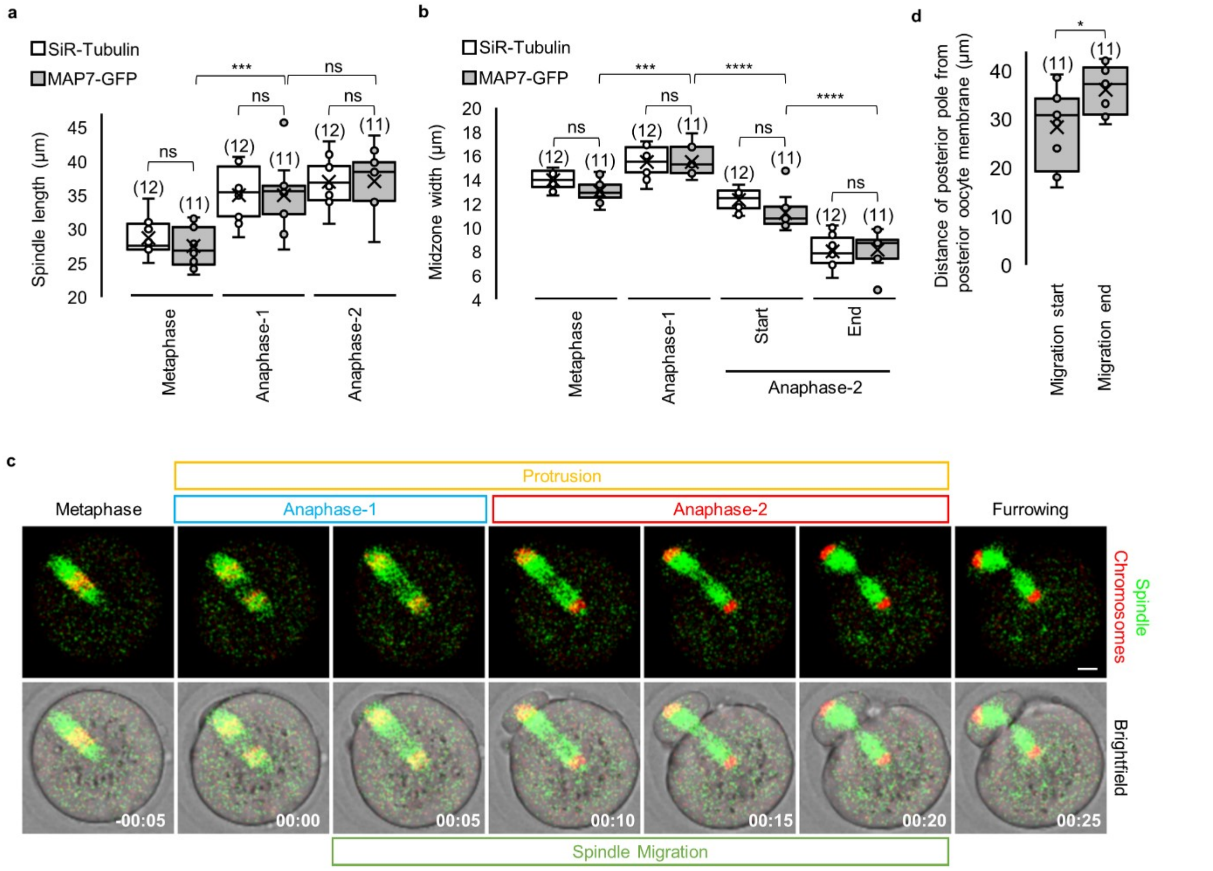

Supplementary Figure 2.

Characteristics of anaphase and spindle migration for MAP7-GFP-labelled spindles. (**a**, **b**) Quantification of stage-specific spindle dimensions in live oocytes expressing MAP7-GFP. Shown for comparison are measurements for spindles labelled with SiR-Tubulin derived from Fig. 1b, c. (**c**) Shown is a panel of images comprised of selected brightfield and fluorescence frames from a representative timelapse series of an oocyte expressing H2B-RFP and MAP7-GFP. Note that as with SiR-tubulin-labelled spindles (see Fig. 1f), protrusion began following anaphase-onset. (**d**) Quantification of total posterior pole displacement between anaphase-onset and anaphase-completion in MAP7-GFP-expressing oocytes. Times are shown as hours:minutes relative to anaphase-onset. Scale bar, 10 µm. Box plots depict median (horizontal line), mean (crosses), 25th and 75th percentiles (boxes) and 5th and 95th percentiles (whiskers). Oocyte numbers are shown in parenthesis from three independent experiments. *P* values were represented as * for $P < 0.05$, ** for $P < 0.01$, *** for $P < 0.001$ and **** for $P < 0.0001$, ns denoted $P > 0.05$. Statistical comparisons were made using either a two-tailed Student's *t*-test in (d) or one-way ANOVA in (a) and (b). See also Supplementary Movie 2.

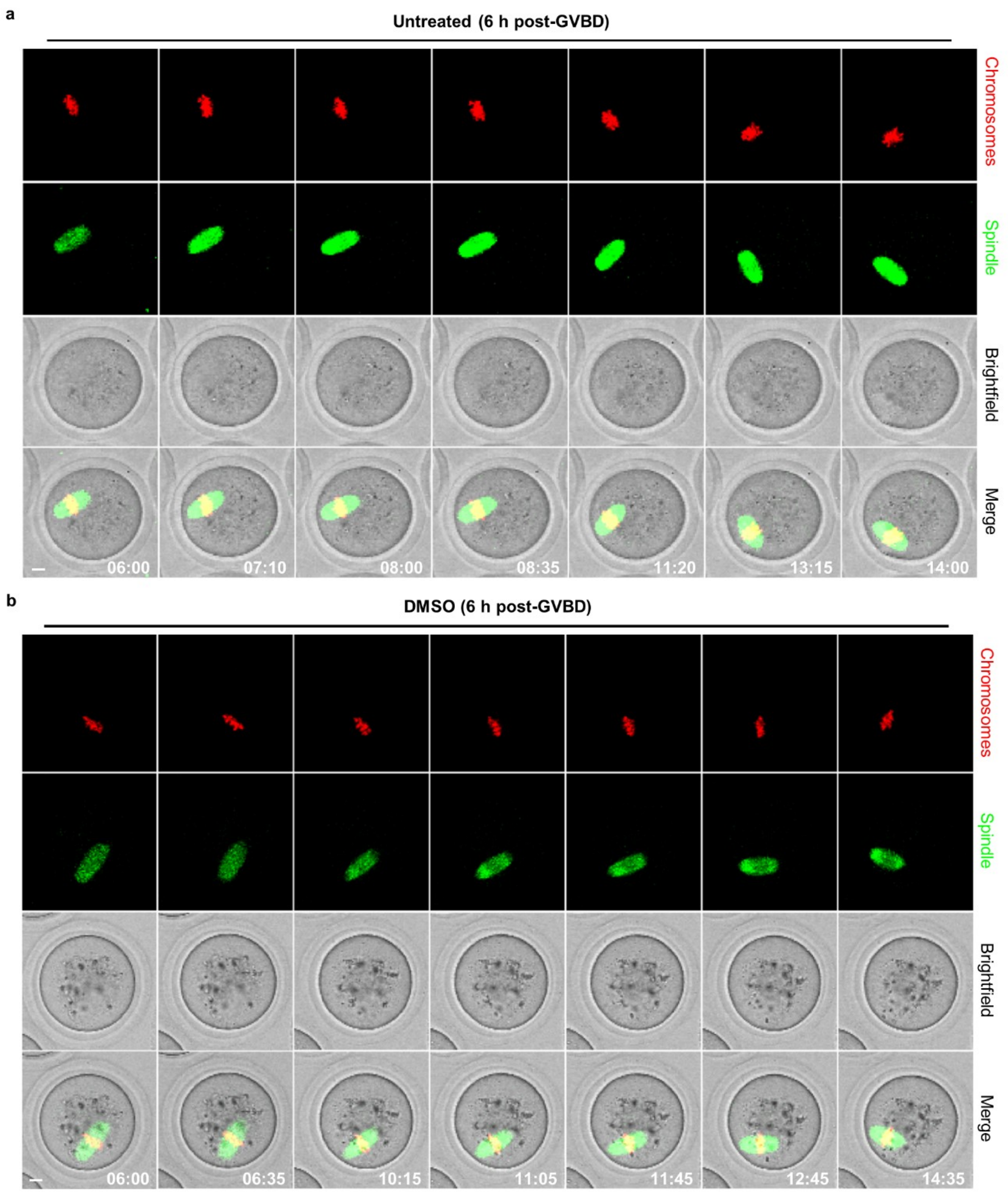

Supplementary Figure 3.

Cortically located metaphase I spindles do not induce protrusion. Here we analysed the small proportion of oocytes (n = 25 per group) from three replicate experiments in which, anaphase did not occur despite bipolar spindle assembly, chromosome alignment and spindle migration to the cortex. (**a**, **b)** Shown are panels of images comprised of selected brightfield and fluorescence frames from representative timelapse series of untreated (a) and 0.2% DMSO-treated (b) oocytes, which assembled bipolar spindles that migrated to the cortex but did not undergo anaphase. Times are shown as hours:minutes relative to 6 h post-GVBD. Scale bar, 10 µm. Note that no protrusion occurs even after the metaphase I spindle has been present at the cortex for extended periods of over 6-8 h. See also Supplementary Movies 4 and 5.

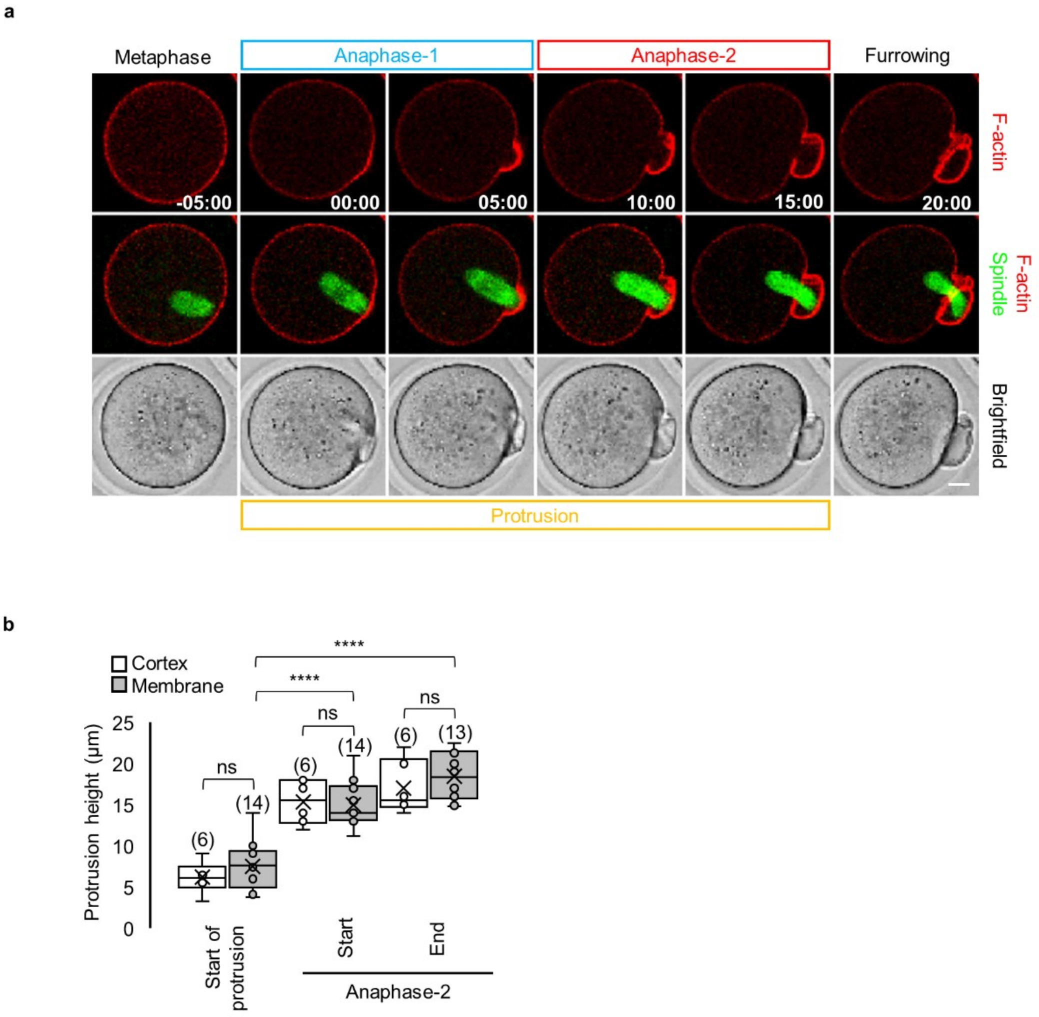

Supplementary Figure 4.

The cortex undergoes protrusion following anaphase-onset. (a) Shown is a panel of images comprised of selected brightfield and fluorescence frames from a representative timelapse series of an oocyte expressing UtrCH-mCherry and SiR-Tubulin depicting cortical protrusion following anaphase-onset. Note that the leading spindle pole occupies the protrusion. (**b**) Quantification of stage-specific heights of cortical and membrane protrusions. Times are shown as hours:minutes relative to anaphase-onset. Scale bar, 10 μm. Box plots depict median (horizontal line), mean (crosses), 25th and 75th percentiles (boxes) and 5th and 95th percentiles (whiskers). Oocyte numbers are shown in parenthesis from three independent experiments. *P* values were represented as * for $P < 0.05$, ** for $P < 0.01$, *** for $P < 0.001$ and **** for $P < 0.0001$, ns denoted $P > 0.05$. Statistical comparisons were made using one-way ANOVA in (b). See also Supplementary Movie 6.

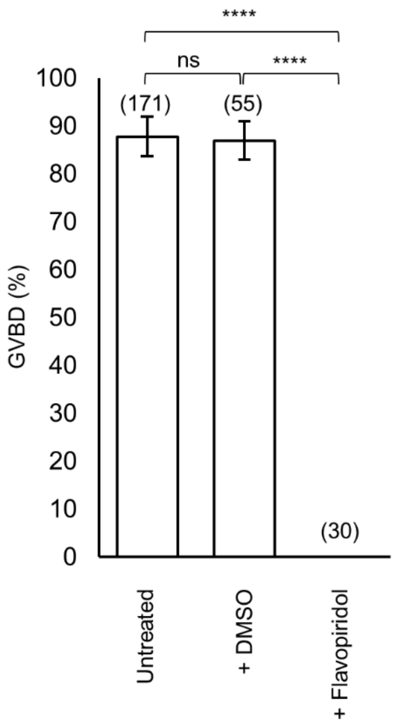

Supplementary Figure 5.

Flavopiridol blocks GVBD indicative of robust Cdk1 inhibition. Shown are the proportions of oocytes that underwent GVBD 3 hours following release from IBMX. Since Cdk1 activation is required for GVBD, the complete absence of GVBD confirms that 5µM Flavopiridol robustly inhibits Cdk1 under our experimental conditions. Data in graphs are presented as the mean ± SEM. Oocyte numbers are shown in parenthesis from three independent experiments. P values were represented as * for $P < 0.05$, ** for $P < 0.01$, *** for $P < 0.001$ and **** for $P < 0.0001$, ns denoted $P > 0.05$. Statistical comparisons were made using one-way ANOVA.

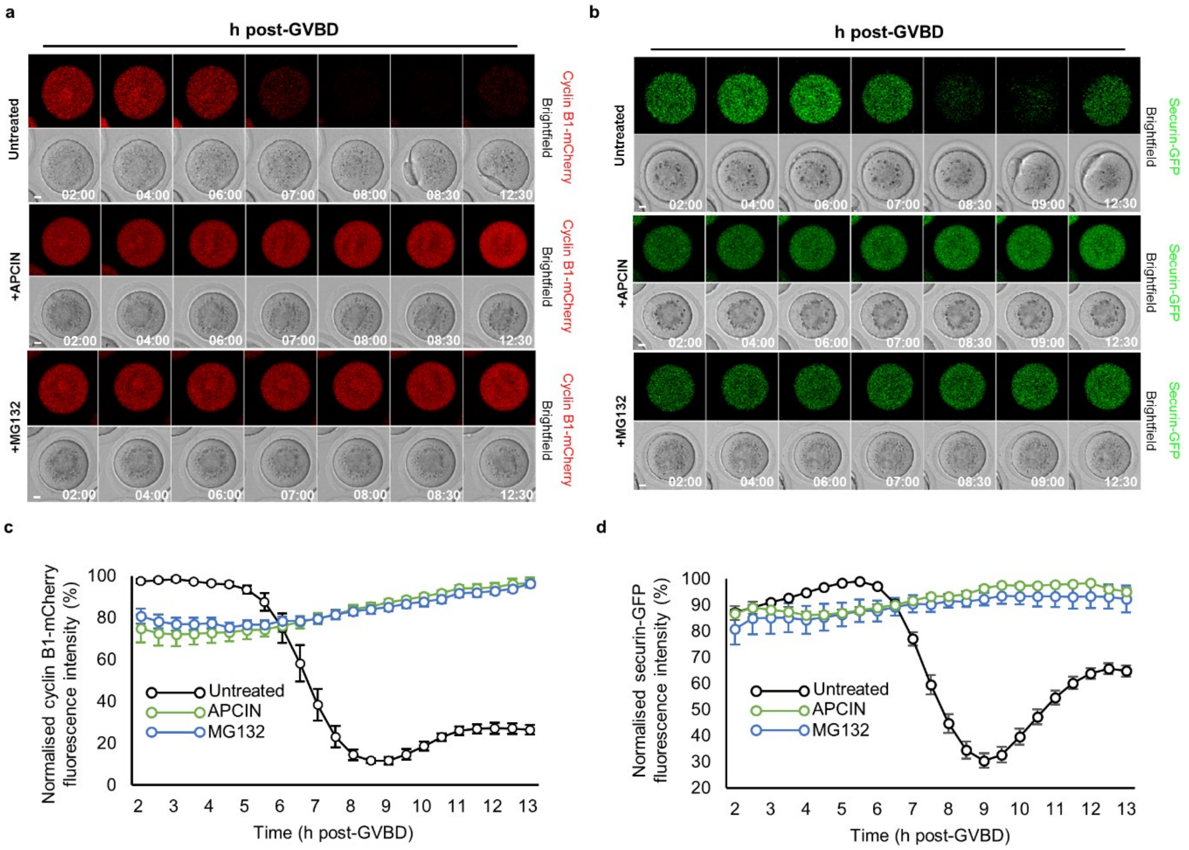

Supplementary Figure 6.

APCIN and MG132 completely inhibit proteolysis. At 2 h post-GVBD, oocytes were transferred to untreated media (n = 31) or to media treated with either 150 µM of the selective APC-Cdc20 inhibitor, APCIN (n = 10) or 5 µM of the 26S proteasome inhibitor, MG132 (n = 16), and immediately transferred to the microscope stage for image acquisition. (**a**, **b**) Shown are panels of images comprised of selected brightfield and fluorescence frames from representative timelapse series of oocytes expressing either cyclin B1-mCherry (a) or securin-GFP (b). Times are shown as hours:minutes relative to GVBD. Scale bar, 10 µm. (**c**, **d**) Fluorescence intensities of cyclin B1-mCherry (c) and securin-GFP (d) were normalised against maximal intensities and plotted over time. Data are presented as the mean ± SEM. Data in (c) and (d) are shown from three independent experiments.

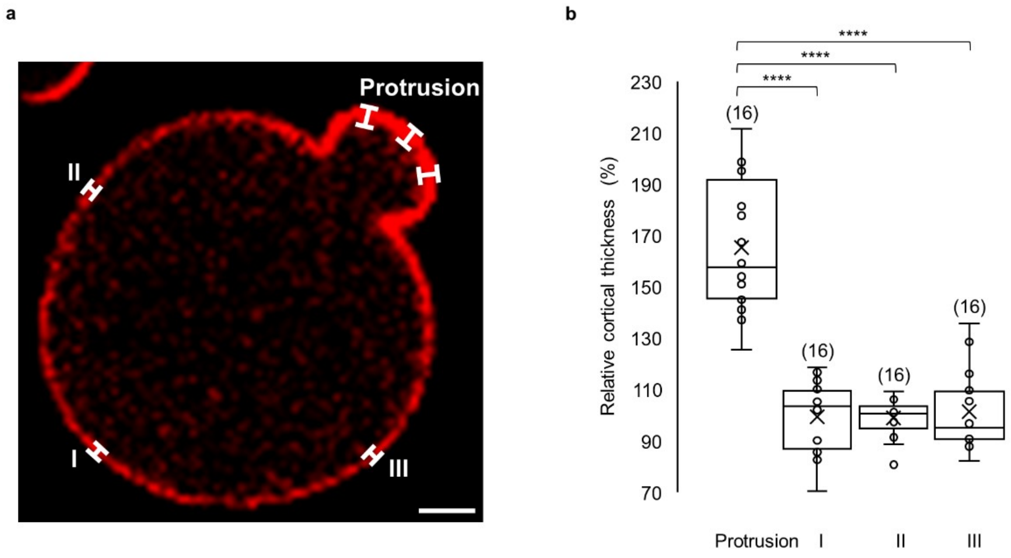

Supplementary Figure 7.

Cortical F-actin thickness is greatest within the protrusion. Cortical thickness was measured at the site of the protrusion and at three other sites at the cortex away from the protrusion in oocytes in which F-actin was labelled using UtrCH-mCherry. (**a**) Method for measuring cortical F-actin thickness. Shown is one z-plane of the UtrCH-mCherry signal for a typical oocyte with a protrusion. Cortical thickness was measured at three sites spanning the leading protrusion surface from which, the mean cortical thickness of the protrusion was determined. For comparison, cortical thickness was measured at three sites away from the protrusion: at the cortex opposite from the protrusion (I) and then at two additional sites between I and the protrusion that were roughly opposite from one another (II and III). Scale bar, 10 µm. (**b**) Relative cortical F-actin thickness at the protrusion and at positions I, II and III. Values shown are relative to the mean of the thicknesses at positions I, II and III for each oocyte. Box plots depict median (horizontal line), mean (crosses), 25th and 75th percentiles (boxes) and 5th and 95th percentiles (whiskers). Oocyte numbers are shown in parenthesis from three independent experiments. *P* values were represented as * for $P < 0.05$, ** for $P < 0.01$, *** for $P < 0.001$ and **** for $P < 0.0001$, ns denoted $P > 0.05$. Statistical comparisons were made using one-way ANOVA in (b).

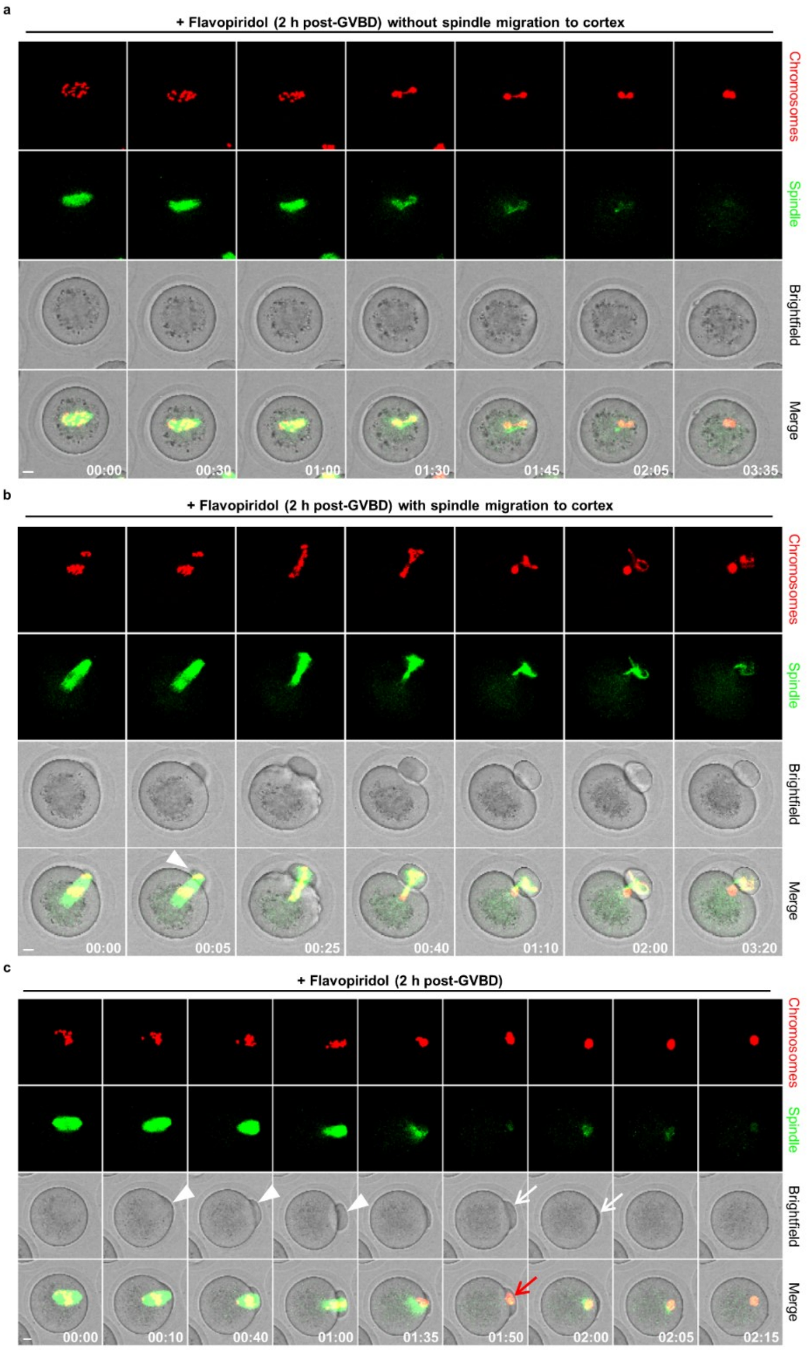

Supplementary Figure 8.

Persistence of a spindle is necessary for sustained protrusion. At 2 h post-GVBD, oocytes were treated with flavopiridol and imaging was commenced. (**a**) Flavopiridol caused spindle migration to the cortex in most cases (71.4 %, n = 28, at 30 min post-treatment) but no protrusion occurred if the spindle collapsed prior to reaching the cortex. (**b**) Sustained protrusions (white arrowhead) followed by furrowing occurred if the spindle migrated to the cortex and advanced into the membrane. (**c**) Protrusions (white arrowheads) receded (white arrows) if the spindle collapsed after protrusion began but prior to completion of furrowing despite the presence of chromosomes within the protrusion (red arrow). Times are shown as hours:minutes relative to 2 h post-GVBD. Scale bar, 10 µm. See also Supplementary Movies 18-20.

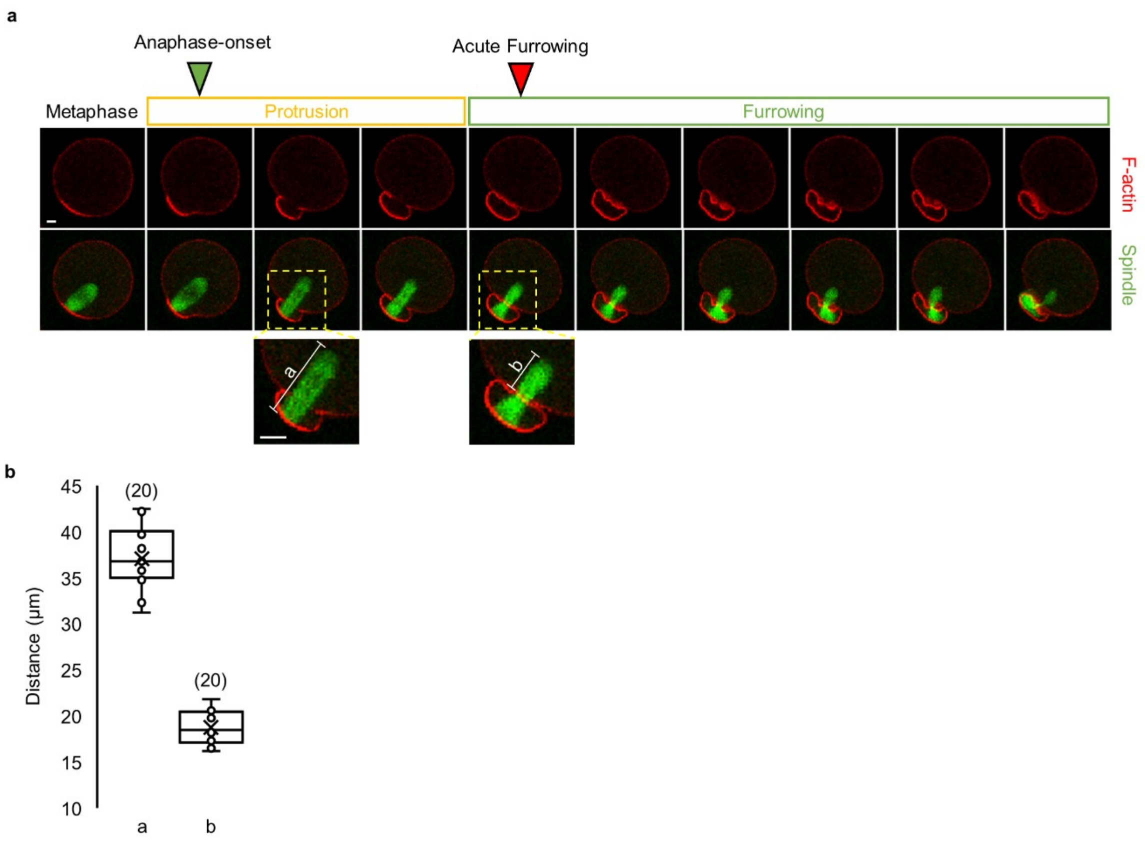

Supplementary Figure 9.

The site of furrowing is defined by the midzone after anaphase spindle migration. (**a**) Shown is a panel of images comprised of selected fluorescence frames from a representative timelapse series of an oocyte expressing UtrCH-mCherry (F-actin) and SiR-Tubulin (microtubules) showing that cortical furrowing occurs roughly halfway along the length of the anaphase spindle. Shown below the panel are magnified images of the boxed regions in which, the measurements a (denoting maximal anaphase spindle length) and b (denoting the length of spindle within the oocyte at furrowing) can more easily be seen. Scale bar, 10 µm. (**b**) Quantification of a and b from live oocytes. Note that b is roughly half the length of a. Box plots depict median (horizontal line), mean (crosses), 25th and 75th percentiles (boxes) and 5th and 95th percentiles (whiskers). Oocyte numbers are shown in parenthesis from three independent experiments. See also Supplementary Movie 25.

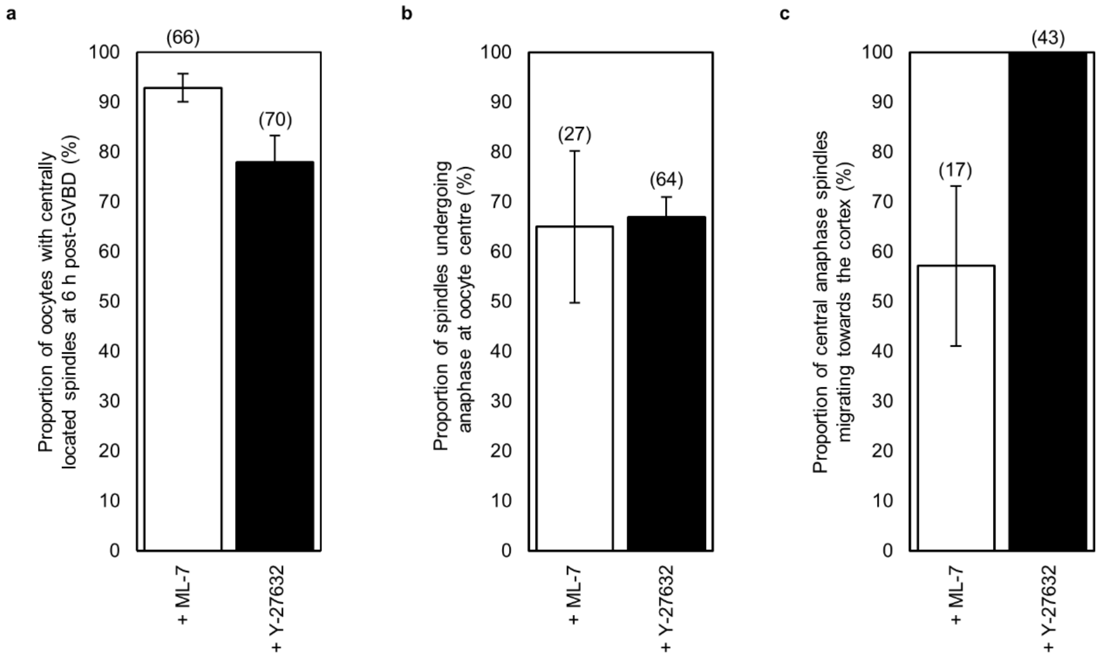

Supplementary Figure 10.

Post-anaphase spindle migration after inhibition of pre-anaphase spindle migration. At 2 h post-GVBD oocytes were treated with either 30 μM of the selective myosin light chain kinase inhibitor, ML-7, or 50 μM of the selective ROCK inhibitor, Y-27632, before imaging was commenced at 6 h post-GVBD for at least 12 h. (**a**) The proportion of oocytes with spindles located at the oocyte centre were determined at the commencement of imaging (6 h post-GVBD). Note that the overwhelming majority of inhibitor-treated oocytes have centrally located spindles at 6 h post-GVBD in contrast with controls (see Supplementary Fig. 1), confirming that inhibition of myosin prevents pre-anaphase spindle migration. (**b**) Proportions of inhibitor-treated oocytes with centrally located spindles that underwent anaphase at the oocyte centre. (**c**) Proportions of oocytes whose spindles underwent anaphase at the oocyte centre and then migrated towards the cortex. Data in graphs are presented as the mean ± SEM. Oocyte numbers are shown in parenthesis from three independent experiments.

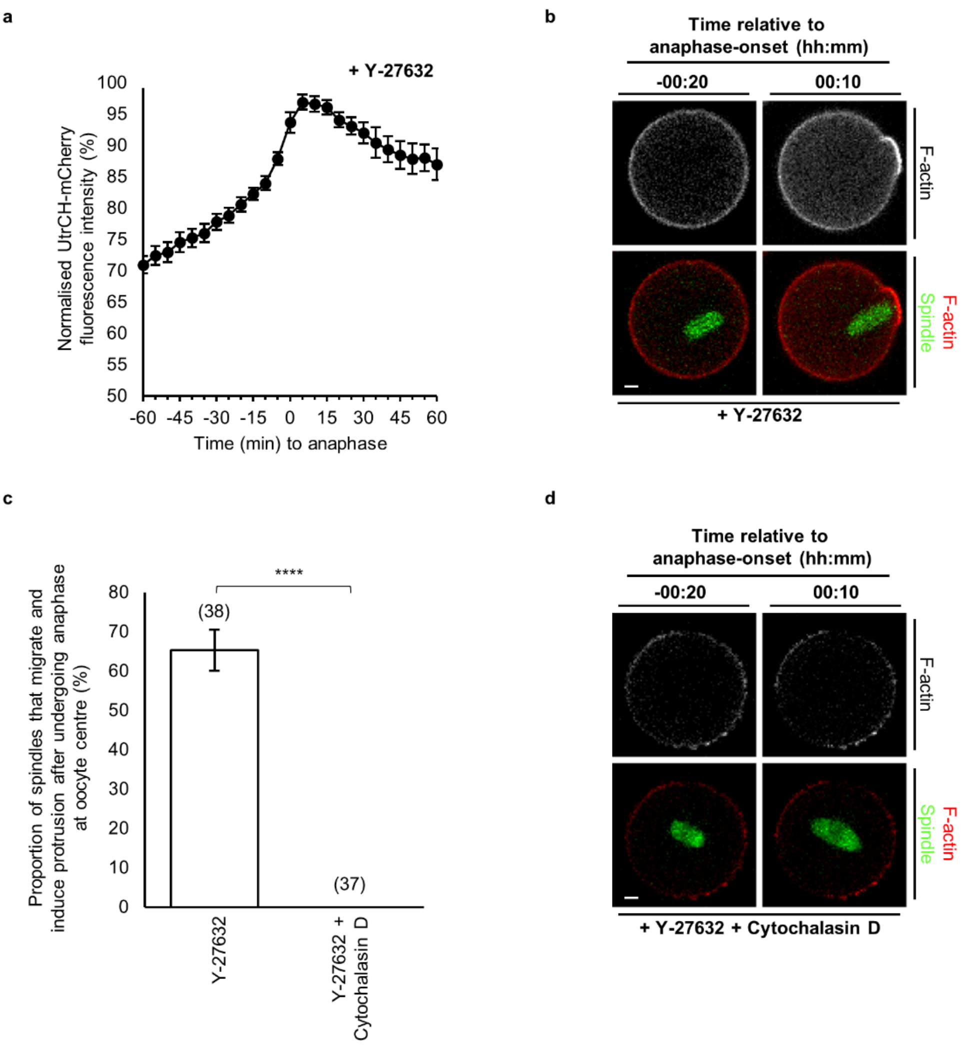

Supplementary Figure 11.

Post-anaphase-onset spindle migration requires cytoplasmic F-actin polymerisation. (**a**) At 2 h post-GVBD, oocytes were treated with Y-27632 and imaging was commenced at 6 h post-GVBD. Cytoplasmic UtrCH-mCherry intensity was quantified relative to anaphase-onset (n = 12). (**b**) Shown is a panel of images comprised of selected fluorescence frames from a representative timelapse series of an oocyte expressing UtrCH-mCherry (F-actin) and SiR-Tubulin (microtubules). (**c**, **d**) The above experiment was repeated but this time, oocytes were transferred to media treated with cytochalasin D at 6 h post-GVBD after spindles had typically migrated to the cortex (see Supplementary Fig. 1b). Proportions of oocytes that underwent migration and protrusion post-anaphase-onset in Y-27632-treated and Y-27632 + cytochalasin D-treated oocytes (c). Shown is a panel of images comprised of selected fluorescence frames from a representative timelapse series of an oocyte expressing UtrCH-mCherry (F-actin) and SiR-Tubulin (microtubules) (d). Times are shown as hours:minutes relative to anaphase-onset. Scale bar, 10 µm. Data in graphs are presented as the mean ± SEM. Oocyte numbers are shown in parenthesis from three independent experiments in (c). *P* values were represented as * for $P < 0.05$, ** for $P < 0.01$, *** for $P < 0.001$ and **** for $P < 0.0001$, ns denoted $P > 0.05$. Statistical comparison was made using a two-tailed Student's *t*-test in (c).

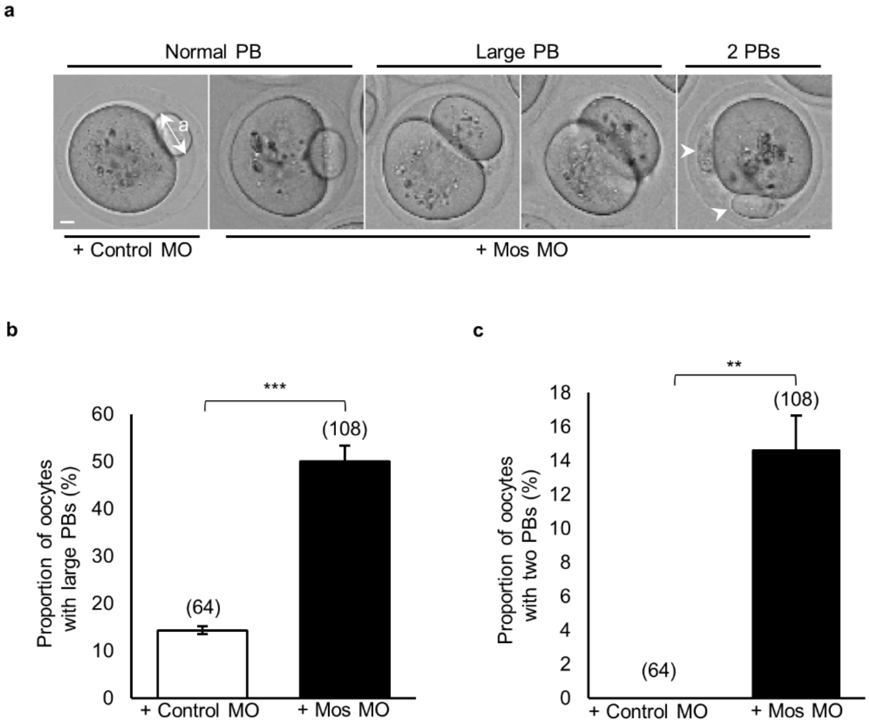

Supplementary Figure 12.

The *MOS*-targeting morpholino induces large and multiple PBs. Mos- and mock-depleted oocytes were allowed to mature *in vitro* for > 20 h, following which oocyte morphology was determined using brightfield imaging. (**a**) Shown is a panel of brightfield images illustrating the phenotypes produced following injection of Mos MO. 2 PBs, oocytes with two polar bodies (white arrowheads); a, diameter of PB. (**b**) Proportion of oocytes with large polar bodies (defined as a > 30 µm). (**c**) Proportion of oocytes with two PBs. Scale bar, 10 µm. Data in graphs are presented as the mean ± SEM. Oocyte numbers are shown in parenthesis from three independent experiments in (b) and (c). P values were represented as * for $P < 0.05$, ** for $P < 0.01$, *** for $P < 0.001$ and **** for $P < 0.0001$, ns denoted $P > 0.05$. Statistical comparisons were made using a two-tailed Student's t-test in (b) and (c).
