# Supplementary material for: Cdk1 inactivation induces post-anaphase-onset spindle migration and membrane protrusion required for extreme asymmetry in mouse oocytes
Source: Nat Commun. 2018 Oct 2;9:4029. doi: 10.1038/s41467-018-06510-9 (PMC6168559; doi:10.1038/s41467-018-06510-9)
Supplement: Supplementary file 2 — Description of Additional Supplementary Files [file 41467_2018_6510_MOESM2_ESM.pdf]

Description of Additional Supplementary Files

File Name: Supplementary Movie 1.
Description: Meiotic maturation, acentrosomal spindle assembly and pre-anaphase spindle migration in mouse oocytes. Movie corresponding to Supplementary Figure 1a showing an oocyte expressing H2B-RFP (red, chromosomes) and treated with SiR-Tubulin (green, spindle) illustrating acentrosomal spindle assembly, pre-anaphase spindle migration, anaphase, PBE and establishment of MII arrest during meiotic maturation. Time is shown as hh:mm relative to GVBD.

File Name: Supplementary Movie 2.
Description: Spindle dynamics during anaphase with microtubules labelled with MAP7- GFP. Movie corresponding to Supplementary Figure 2 showing an oocyte expressing H2BRFP (red, chromosomes) and MAP7-GFP (green, spindle), illustrating spindle elongation during anaphase-1, subsequent spindle midzone narrowing in anaphase-2 and protrusion occurring in relation to anaphase-onset and spindle migration. Time is shown in hh:mm relative to anaphase-onset.

File Name: Supplementary Movie 3.
Description: Spindle migration into the membrane occurs after anaphase-onset and induces membrane protrusion. Movie corresponding to Figure 1f showing that membrane protrusion begins shortly after anaphase-onset and increases in size as anaphase progresses and the spindle migrates away from the posterior membrane, visualised by simultaneously imaging the entire oocyte in the brightfield channel, H2B-RFP (red, chromosomes) and treated with SiR-Tubulin (green, spindles). Time is shown as hh:mm relative to anaphaseonset.

File Name: Supplementary Movie 4.
Description: Membrane protrusion does not occur in association with cortically-located metaphase spindles and chromosomes in untreated control oocytes. Movie corresponding to Supplementary Figure 2a showing that membrane protrusion does not occur even after the metaphase spindle (green, labelled using SiR-Tubulin) and chromosomes (red, H2B-RFP) is present at the cortex for several hours. Note as well that protrusion does not occur even after the spindle rotates to bring the metaphase chromosomes closer to the cortex. Time is shown as hh:mm from 6 h post-GVBD.

File Name: Supplementary Movie 5.
Description: Membrane protrusion does not occur in association with cortically-located metaphase spindles in DMSO-treated control oocytes. Movie corresponding to Supplementary Figure 2b illustrating an oocyte expressing H2B-RFP (red, chromosomes) and treated with SiR-Tubulin (green, spindle), displaying identical findings as observed with the untreated control oocyte in Movie 3. Since inhibitors used in other experiments were dissolved in DMSO, DMSO-treatment served as another control group alongside untreated oocytes. Time is shown as hh:mm from 6 h post-GVBD.

File Name: Supplementary Movie 6.
Description: Spindle migration into the membrane occurs after anaphase-onset and induces protrusion of the cortex akin to protrusion observed with membranes. Movie corresponding to

Supplementary Figure 4 showing that anaphase-onset – which is clearly identifiable by the appearance of the low-intensity midzone and the increase in spindle length – is followed shortly after by protrusion of the cortex. Here the cortex is labelled using UtrCH-mCherry (red) whilst the spindle is labelled with SiR-Tubulin (green). Time is shown as hh:mm relative to anaphase-onset.

File Name: Supplementary Movie 7.
Description: Timing of anaphase-onset and protrusion relative to GVBD in untreated oocytes. Movie corresponding to Figure 2a showing an oocyte expressing H2B-RFP (red, chromosomes) and treated with SiR-Tubulin (green, spindle), illustrating that anaphase-onset and protrusion occur more than 1 h after the start of imaging, that is, more than 7 h postGVBD. Time is shown as hh:mm relative to GVBD.

File Name: Supplementary Movie 8.
Description: Timing of anaphase-onset and protrusion relative to GVBD is unaffected by DMSO treatment. Movie corresponding to Figure 2b showing an oocyte expressing H2BRFP (red, chromosomes) and treated with SiR-Tubulin (green, spindle), illustrating that as with untreated oocytes (see Movie 10), anaphase-onset and protrusion occur more than 1 h following the commencement of imaging at 6 h post-GVBD, that is, more than 7 h postGVBD. Time is shown as hh:mm relative to GVBD.

File Name: Supplementary Movie 9.
Description: Flavopiridol-induced Cdk1 inactivation accelerates anaphase-onset and protrusion. Movie corresponding to Figure 2c showing an oocyte expressing H2B-RFP (red, chromosomes) and treated with SiR-Tubulin (green, spindle), illustrating that anaphase and protrusion are already apparent by 10 min after treatment with flavopiridol, markedly faster than in controls (compare with Supplementary Movies 7 and 8; see Figure 2e). Time is shown as hh:mm relative to GVBD.

File Name: Supplementary Movie 10.
Description: Flavopiridol-induced Cdk1 inactivation induces spindle migration and protrusion in the absence of proteolysis and anaphase. Movie corresponding to Figure 2f showing an oocyte expressing H2B-RFP (red, chromosomes), treated with SiR-Tubulin (green, spindle) and treated with flavopiridol at 4 h post-GVBD, which is prior to onset of proteolysis (see Supplementary Figure 6). Note that flavopiridol rapidly induces spindle migration and protrusion within 25-30 min and that, in the absence of anaphase, chromosomes become trapped within the cleavage furrow. Time is shown as hh:mm relative to GVBD.

File Name: Supplementary Movie 11.
Description: Flavopiridol-induced Cdk1 inactivation induces spindle migration and protrusion even when APC-mediated proteolysis is inhibited. Movie corresponding to Figure 2g showing an oocyte expressing H2B-RFP (red, chromosomes), treated with SiRTubulin (green, spindle) and treated with APCIN (from 4 h post-GVBD) and flavopiridol (from 6 h post-GVBD). Note that spindle migration and protrusion occur shortly after flavopiridol treatment and that due to lack of proteolysis, chromosomes do not separate and become trapped within the cleavage furrow akin to the oocyte in Movie 10. Time is shown as hh:mm relative to GVBD.

File Name: Supplementary Movie 12.
Description: Flavopiridol-induced Cdk1 inactivation induces spindle migration and protrusion even when proteolysis is inhibited using MG132. Movie corresponding to Figure 2h showing an oocyte expressing H2B-RFP (red, chromosomes), treated with SiRTubulin (green, spindle) and treated with MG132 (from 4 h post-GVBD) and flavopiridol (from 6 h post-GVBD). Note that as in Movies 10 and 11, spindle migration and protrusion occur shortly after flavopiridol treatment and that due to lack of proteolysis, chromosomes do not separate and become trapped within the cleavage furrow. Time is shown as hh:mm relative to GVBD.

File Name: Supplementary Movie 13.
Description: Protrusion and PBE occur following anaphase-onset in DMSO-treated oocytes in which the spindle remains intact. Movie corresponding to Figure 3b showing an oocyte expressing H2B-RFP (red, chromosomes), treated with SiR-Tubulin (green, spindle) and treated with DMSO from 6 h post-GVBD in which, anaphase and protrusion begin more than an hour following the start of imaging as observed before (see Movies 7 and 8). Time is shown as hh:mm relative to GVBD.

File Name: Supplementary Movie 14.
Description: Chromosomes alone after nocodazole treatment cannot induce sustained protrusions. Movie corresponding to Figure 3c showing an oocyte expressing H2B-RFP (red, chromosomes) and treated with SiR-Tubulin (green, spindle) that was treated with nocodazole from 2 h post-GVBD before imaging began at 6 h post-GVBD. Note that the spindle is absent and chromosomes are located at the cortex. Transient small protrusions occur in relation to the chromosomes but despite several hours of imaging there are no sustained protrusions. Time is shown as hh:mm relative to GVBD.

File Name: Supplementary Movie 15.
Description: Flavopiridol treatment induces accelerated anaphase and protrusion in the presence of a spindle. Movie corresponding to Figure 3d showing an oocyte expressing H2B-RFP (red, chromosomes) and treated with SiR-Tubulin (green, spindle) that was treated with flavopiridol immediately prior to commencing imaging at 6 h post-GVBD. Note that as before (see Movie 9), flavopiridol rapidly (within 10-15 min) induced anaphase and protrusion. Time is shown as hh:mm relative to GVBD.

File Name: Supplementary Movie 16.
Description: Chromosomes alone cannot induce sustained protrusions even following forced Cdk1 inactivation with flavopiridol. Movie corresponding to Figure 3e showing an oocyte expressing H2B-RFP (red, chromosomes) and treated with SiR-Tubulin (green, spindle) that was treated with nocodazole from 2 h post-GVBD followed by flavopiridol at 6 h post-GVBD immediately before commencement of imaging. Note that the spindle is absent and chromosomes are located close to the cortex but no protrusions form. Chromosomes eventually drift towards the oocyte centre and decondense. Time is shown as hh:mm relative to GVBD.

File Name: Supplementary Movie 17.
Description: Allowing the spindle to reassemble following nocodazole-washout enables protrusion and PBE following flavopiridol treatment. Movie corresponding to Figure 3f showing an oocyte

expressing H2B-RFP (red, chromosomes) and treated with SiR-Tubulin (green, spindle) that was treated with nocodazole from 2-6 h post-GVBD before being washed from nocodazole and treated with flavopiridol immediately prior to imaging. Note that a bipolar spindle reassembles following washout from the spindle inhibitor and immediately migrates into the membrane to induce protrusion and PBE. Time is shown as hh:mm relative to GVBD.

File Name: Supplementary Movie 18.
Description: If the spindle collapses following flavopiridol treatment, chromosomes alone do not induce sustained protrusions. Movie corresponding to Supplementary Figure S8a showing an oocyte expressing H2B-RFP (red, chromosomes) and treated with SiR-Tubulin (green, spindle) that was treated with flavopiridol at 2-3 h post-GVDB, that is, before spindle migration to the cortex (see Supplementary Figure 1). Note that the spindle commences migration but collapses before reaching the cortex and no sustained protrusion forms akin to observations with nocodazole treatment (see Movies 14 and 16). Time is shown as hh:mm relative to 2 h post-GVBD.

File Name: Supplementary Movie 19.
Description: Protrusion and PBE occur if the spindle remains intact following flavopiridol treatment and migrates into the cortex. Movie corresponding to Supplementary Figure S8b showing an oocyte expressing H2B-RFP (red, chromosomes) and treated with SiR-Tubulin (green, spindle) that was treated with flavopiridol from 2-3 h post-GVBD as in Movie 18 but in this case, the spindle remained intact long enough to migrate into the cortex resulting in protrusion. Because flavopiridol treatment occurred prior to proteolysis, no anaphase occurred and chromosomes became trapped within the cleavage furrow. Time is shown as hh:mm relative to 2 h post-GVBD.

File Name: Supplementary Movie 20.
Description: The protrusion recedes if the spindle collapses prior to completion of PBE. Movie corresponding to Supplementary Figure S8c showing an oocyte that was treated with flavopiridol from 2 h post-GVBD showing the spindle remain intact long enough to migrate into the cortex and create a protrusion but then collapse prior to completion of PBE resulting in protrusion regression. Time is shown as hh:min from 2 h post-GVBD.

File Name: Supplementary Movie 21.
Description: Cytoplasmic F-actin levels undergo a marked increase contemporaneously with anaphase-onset. Movie corresponding to Figure 4b showing an oocyte expressing UtrCH-mCherry (monochrome, F-actin) and treated with SiR-Tubulin (green, spindle) illustrating changes in cytoplasmic F-actin intensity over time. Note that the UtrCH-intensity increases post-anaphase-onset concurrently with spindle migration and protrusion. Time is shown as hh:mm relative to anaphase-onset.

File Name: Supplementary Movie 22.
Description: Cytoplasmic F-actin levels increase following flavopiridol treatment. Movie corresponding to Figure 4e showing an oocyte expressing UtrCH-mCherry (monochrome, Factin) and treated with SiR-Tubulin (green, spindle) that was treated with flavopiridol at 4 h post-GVBD. Note the change in oocyte position when flavopiridol was added during imaging and the increase in

UtrCH-intensity shortly thereafter. Time is shown as hh:mm relative to flavopiridol addition during imaging.

File Name: Supplementary Movie 23.
Description: Treatment with cytochalasin D prevents the cytoplasmic F-actin increase associated with anaphase. Movie corresponding to Figure 4g showing an oocyte expressing UtrCH-mCherry (monochrome, F-actin) and treated with SiR-Tubulin (green, spindle) that was treated with cytochalasin D at 6 h post-GVBD. Note that although anaphase occurs whilst the spindle is located adjacent to the cortex, the cytoplasmic UtrCH-intensity does not increase and no spindle migration or protrusion occurs (compare with Supplementary Movie 21; see Figure 4b). Time is shown as hh:mm relative to anaphase-onset.

File Name: Supplementary Movie 24.
Description: Treatment with CK666 prevents cortical F-actin changes but does not impact anaphase-associated cytoplasmic F-actin increase and accompanying spindle migration and protrusion. Movie corresponding to Figure 5d showing an oocyte expressing UtrCHmCherry (monochrome, F-actin) and treated with SiR-Tubulin (green, spindle) that was treated with CK666 at 6 h post-GVBD. Note that although the anaphase-associated increase in cortical F-actin is blunted, spindle migration and protrusion remain intact. Time is shown as hh:mm relative to anaphase-onset.

File Name: Supplementary Movie 25.
Description: Cortical furrowing occurs halfway along the length of the anaphase spindle. Movie corresponding to Supplementary Figure 9a showing that the ingressing margins of the cortical furrow come together with one another midway along the length of the anaphase spindle. Here the cortex is labelled using UtrCH-mCherry (red) whilst the spindle is labelled with SiR-Tubulin (green). As in Movie 6, note that anaphase-onset is clearly identifiable by the appearance of the low-intensity midzone and the increase in spindle length and is followed shortly after by protrusion of the cortex and migration of the spindle away from the posterior cortex. Time is shown as hh:mm relative to anaphase-onset.

File Name: Supplementary Movie 26.
Description: Near-symmetrical cleavage occurs if spindle migration does not occur after anaphase-onset occurs at the oocyte centre. Movie corresponding to Figure 6c showing an ML-7-treated oocyte expressing H2B-RFP (red, chromosomes) and treated with SiR-Tubulin (green, spindle) in which anaphase occurs close to the centre of the oocyte. Note that the anaphase spindle does not migrate and that the furrow ingresses over the spindle midzone leading to cleavage. Note as well that the spindle midzone is slightly closer to the upper oocyte membrane and that furrowing commences earlier at this membrane. Time is shown as hh:mm relative to anaphase-onset.

File Name: Supplementary Movie 27.
Description: Spindle migration after anaphase-onset occurs at the oocyte centre generates asymmetry. Movie corresponding to Figure 6a showing an ML-7-treated oocyte expressing H2B-RFP (red, chromosomes) and treated with SiR-Tubulin (green, spindle) in which anaphase occurs close to the centre of the oocyte. Note that unlike the oocyte in Movie 26, here the anaphase spindle does migrate before furrowing leads to cleavage. However, furrowing begins before the leading pole

arrives at the cortex so that protrusion does not occur. The PB in this case is smaller than in Movie 26, but still larger than normal. Time is shown as hh:mm relative to anaphase-onset.

File Name: Supplementary Movie 28.
Description: Spindle migration combined with cortical protrusion after anaphase-onset at the oocyte centre produce small PBs. Movie corresponding to Figure 6c showing an Y27632-treated oocyte expressing H2B-RFP (red, chromosomes) and treated with SiR-Tubulin (green, spindle) in which anaphase progresses to completion at the centre of the oocyte. Strikingly, the anaphase spindle then migrates the complete distance to the cortex where the leading pole induces a protrusion prior to furrowing resulting in production of a normal-sized PB. Time is shown as hh:mm relative to anaphase-onset.

File Name: Supplementary Movie 29.
Description: Mos-depletion results in an elongated anaphase spindle and compromised post-anaphase-onset spindle migration. Movie corresponding to Figure 7b showing a Mosdepleted oocyte expressing H2B-RFP (red, chromosomes) and treated with SiR-Tubulin (green, spindle) in which anaphase occurs close to the cortex of the oocyte. The spindle then undergoes marked elongation and does not migrate to the extent of control oocytes (see Supplementary Movie 3) resulting in a large PB. Time is shown as hh:mm relative to anaphase-onset.

File Name: Supplementary Movie 30.
Description: Typical asymmetry results after perpendicular spindles migrate into the membrane following anaphase-onset leading to displacement of half of the spindle beyond the oocyte. Movie corresponding to Figure 8c showing an untreated oocyte with a spindle adjacent and oriented perpendicular to the cortex (see Figure 8a) that migrates into the membrane following anaphase-onset resulting in cleavage of the PB after half of the spindle has been delivered into the protrusion. Time is shown as hh:mm relative to anaphaseonset.

File Name: Supplementary Movie 31.
Description: In the absence of post-anaphase-onset spindle migration and protrusion, the cleavage furrow forms at the adjacent cortex overlying the midzone resulting in nearsymmetrical cleavage. Movie corresponding to Figure 8d showing an untreated oocyte with a spindle adjacent and oriented non-perpendicular to the cortex (see Figure 8a) that fails to migrate into the membrane following anaphase-onset – therefore not inducing a protrusion – resulting in the furrow tracking over the midzone through the central region of the oocyte. Note as well that the midzone directs furrowing at the adjacent overlying cortex but not at the more distal, contralateral cortex. Time is shown as hh:mm relative to anaphase-onset.

File Name: Supplementary Movie 32.
Description: Furrowing occurs simultaneously at two opposing membranes when the spindle undergoes anaphase at the oocyte centre and its midzone is equidistant from both membranes. Movie corresponding to Figure 8e showing an oocyte having a spindle that undergoes anaphase close to the oocyte centre. Note that the midzone is located roughly equidistant from the overlying cortices and that furrowing occurs relatively symmetrically over both surfaces. This can be

contrasted with the oocyte in Movie 31 in which furrowing initiates at the cortical surface closest to the off-centre midzone. Time is shown as hh:mm relative to anaphase-onset.

File Name: Supplementary Movie 33.
Description: Post-anaphase-onset spindle migration and protrusion enable corticallylocated non-perpendicular spindles to generate asymmetry. Movie corresponding to Figure 8f showing an oocyte with a non-perpendicular spindle initiating anaphase very similar to the oocyte in Movie 31. In contrast to the oocyte in Movie 31, however, here one spindle pole engaged the cortex and the spindle migrated into the membrane resulting in protrusion, and ultimately, in asymmetrical division.
